# Chemical Insights into Oxidative and Nitrative Modifications of DNA

**DOI:** 10.3390/ijms242015240

**Published:** 2023-10-16

**Authors:** Celia María Curieses Andrés, José Manuel Pérez de la Lastra, Celia Andrés Juan, Francisco J. Plou, Eduardo Pérez-Lebeña

**Affiliations:** 1 Hospital Clínico Universitario, Avenida de Ramón y Cajal, 3, 47003 Valladolid, Spain; cmcurieses@gmail.com; 2 Institute of Natural Products and Agrobiology, CSIC-Spanish Research Council, Avda. AstrofísicoFco. Sánchez, 3, 38206 La Laguna, Spain; 3 Cinquima Institute and Department of Organic Chemistry, Faculty of Sciences, Valladolid University, Paseo de Belén, 7, 47011 Valladolid, Spain; celia.andres.juan@uva.es; 4 Institute of Catalysis and Petrochemistry, CSIC-Spanish Research Council, 28049 Madrid, Spain; fplou@icp.csic.es; 5 Sistemas de Biotecnología y Recursos Naturales, 47625 Valladolid, Spain; info@glize.eu

**Keywords:** DNA, oxidative and nitrative DNA damage, inflammation, 2′-deoxyribose oxidation products

## Abstract

This review focuses on DNA damage caused by a variety of oxidizing, alkylating, and nitrating species, and it may play an important role in the pathophysiology of inflammation, cancer, and degenerative diseases. Infection and chronic inflammation have been recognized as important factors in carcinogenesis. Under inflammatory conditions, reactive oxygen species (ROS) and reactive nitrogen species (RNS) are generated from inflammatory and epithelial cells, and result in the formation of oxidative and nitrative DNA lesions, such as 8-oxo-7,8-dihydro-2′-deoxyguanosine (8-oxodG) and 8-nitroguanine. Cellular DNA is continuously exposed to a very high level of genotoxic stress caused by physical, chemical, and biological agents, with an estimated 10,000 modifications occurring every hour in the genetic material of each of our cells. This review highlights recent developments in the chemical biology and toxicology of 2′-deoxyribose oxidation products in DNA.

## 1. Introduction

**Figure 1:**
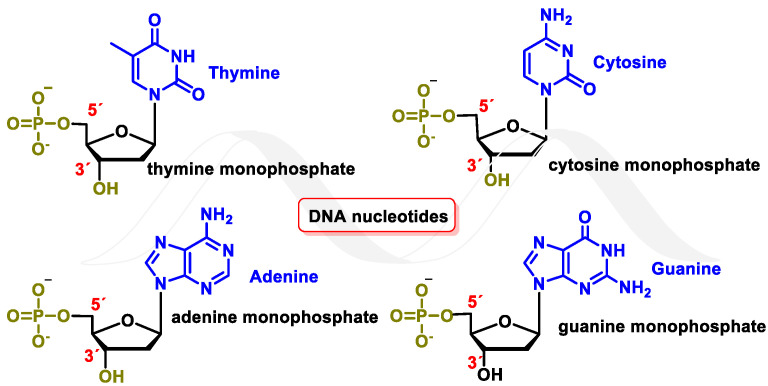
DNA nucleotides.

Deoxyribonucleic acid (DNA) is a macromolecule that contains the genetic information of an organism. It is a biopolymer, with nucleotides being its monomeric units. Each nucleotide consists of a phosphorylated nucleoside. In each nucleoside, the nitrogenous bases guanine (G), cytosine (C), thymine (T), or adenine (A) are connected to a deoxyribose (DNA) molecule by a β-N-glycosidic bond. Nucleotides can be joined by 5′→3′ phosphodiester bonds, forming a DNA strand by phosphodiester or nucleotide bonding, between two consecutive nucleotides. It is an ester-like bond that is established between the phosphate group located at the 5′-position of one nucleotide and the hydroxyl group located at the 3′ carbon of the next nucleotide. The DNA nucleotides are deoxyadenosine-5′-monophosphate (dAMP), deoxyguanosine-5′-monophosphate (dGMP), deoxycytidine-5′-monophosphate (dCMP), and deoxythymidine-5′-monophosphate (dTMP) [1] (Figure 1).

**Figure 2:**
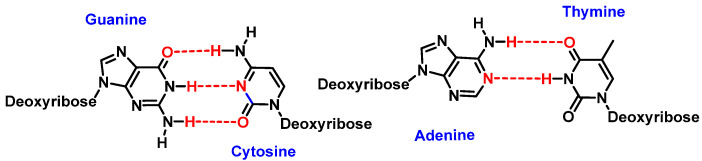
Watson and Crick’s pairing: guanine–cytosine and adenine–thymine.

Watson and Crick’s pairing of nitrogenous bases involves guanine binding to cytosine (G-C) and adenine to thymine (A-T). This bonding takes place through hydrogen bonds where oxygen or nitrogen provides electron density to the hydrogen of the complementary nitrogenous base [2] (Figure 2).

## 2. Causes of DNA Damage

**Figure 3:**
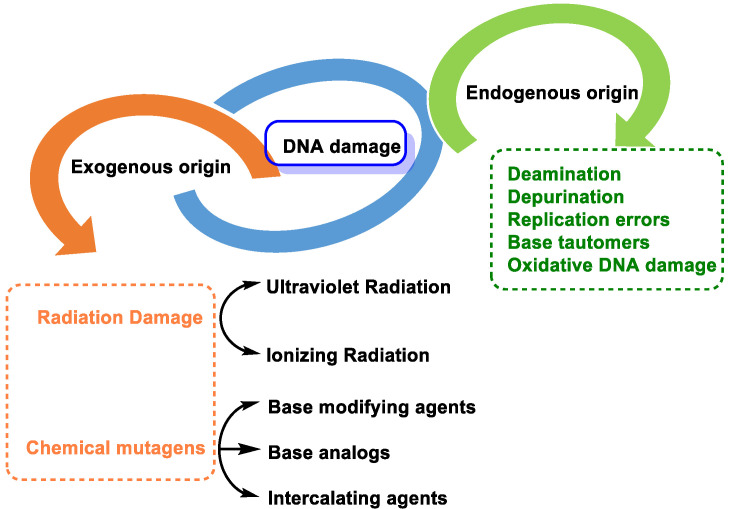
Types of DNA damage.

DNA molecules can be damaged in many ways, and this damage can occur at a rate of 10^4^ to 10^6^ molecular lesions per cell per day. DNA damage can be classified according to the agents that produce them into two main types: (i) endogenous damage that arises naturally, and in the absence of known causative agents, spontaneous DNA lesions are random events [3], and (ii) exogenous damage that occurs in the presence of known causative agents [4] (Figure 3).

Endogenous DNA damage is caused by hydrolytic reaction of DNA with water and by reaction of DNA with ROS and RNS, which are present within cells [5]. Exogenous DNA damage, on the other hand, occurs when environmental, physical, and chemical agents damage DNA, and these agents include UV and ionizing radiation, alkylating agents, and cross-linking agents [6].

### 2.1. DNA Damage by Endogenous Cellular Processes

**Figure 4:**
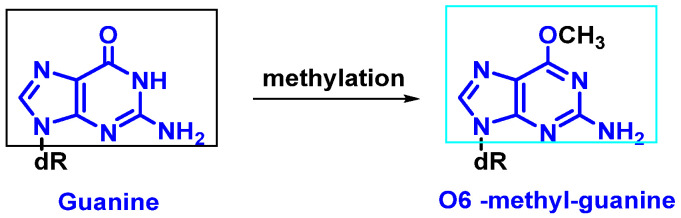
Formation of O6-methyl-guanine.

**Figure 5:**
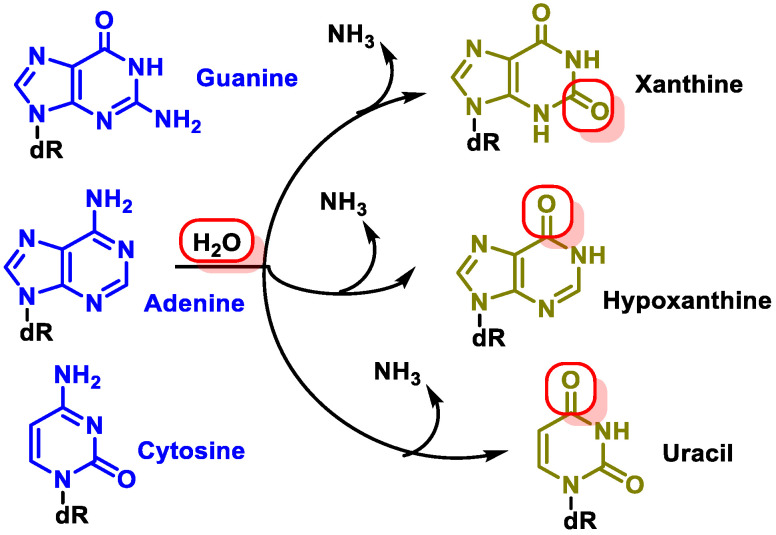
Deamination of adenine, guanine, and cytosine.

It is estimated that each human cell sustains about 70,000 injuries per day. Seventy-five percent of lesions are single-stranded DNA breaks, either due to oxidative damage during cellular metabolism or base hydrolysis [7]. We highlight the damage produced by the following mechanisms:

- Replication errors: DNA polymerase can incorrectly select the nucleotide for insertion into the complementary strand during replication. The substitution of one nucleotide for another is a point mutation, which is the simplest form of DNA damage [8].
- Modification of nitrogenous bases by methylation: Atypical methylation of nitrogenous bases produces thousands of DNA lesions per cell per day. An example is the formation of a methylated derivative of guanine (O6-methyl-guanine). This modified base can generate a post-replicative mutation because it can pair with the same probability with cytosines or thymines [9] (Figure 4).
- Changes in DNA’s nitrogenous bases by deamination: Another type of alteration that DNA undergoes is the loss of amino groups from its nitrogenous bases. Three of the four DNA bases, adenine, guanine, and cytosine, contain amino groups that can be lost in a variety of temperature- and pH-dependent reactions, thereby transforming them into hypoxanthine, xanthine, and uracil [10] (Figure 5).

The most common type of deamination is the spontaneous deamination of cytosine to uracil, which occurs at a rate of about 100 bases/cell/day [11]. Furthermore, adenine deamination is 50 times less likely than cytosine deamination [12].

### 2.2. Loss of Nitrogenous Bases by Depurination or Depyrimidination

**Figure 6:**
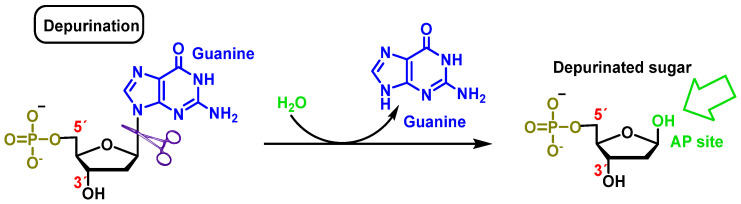
Deoxyguanosine clearance in which the β-N-glycosidic bond is cleaved hydrolytically, thereby releasing guanine.

Cleavage consists of the breakage of the glycosidic bond between the nitrogenous base and the deoxyribose, resulting in the loss of the nitrogenous base from the DNA double helix. The loss of purines (adenine or guanine) or pyrimidines (thymine or cytosine) by breaking the glycosidic bond between the nitrogenous base and the sugar (deoxyribose) generates a site called AP (apurinic or apyrimidinic) [13]. An average of 5000 to 10,000 AP sites can be produced per cell per day. Extreme pH conditions and high temperatures influence their generation [14,15]. Abasic sites are unstable and cause single strand breaks (SSB) (Figure 6).

### 2.3. Base Tautomers

**Figure 7:**
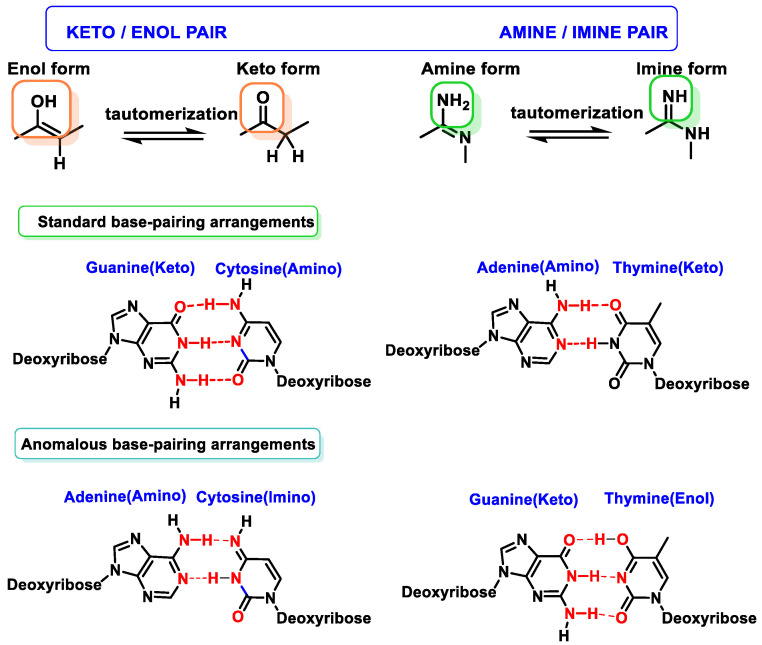
Tautomerism of purine and pyrimidine bases in DNA.

Tautomerism of purine bases in DNA is one of the earliest recognized mechanisms for rationalizing mutations and interconversion of different adenine or guanine tautomers (amino–imino and lactam–lactide forms) and produces mispairing with pyrimidines (which also exhibit tautomeric forms) [16]. The enol/imino forms of DNA bases are rare and tend to cause mispairings, which can result in DNA sequence changes, and often occurs in some types of cancer [17] (Figure 7).

Oxidative damage to DNA is a consequence of cellular metabolism itself and is caused by reactive oxygen species (ROS) and reactive nitrogen species (RNS), which act as strong oxidants reacting with the nitrogenous bases, causing the breakage of one or both DNA strands [18]. One of the main alterations caused by free radicals (FR) is the transformation of guanine into 8-dihydro-deoxyguanine (8-oxo-G), which in DNA replication may pair normally with cytosine or abnormally with adenine, producing transversions (GC to TA) in DNA [19,20]. ROS and RNS also can be produced by macrophages and neutrophils [21] at sites of infection and inflammation [22]. These types of endogenous damage can induce an average of 100,000 lesions per cell per day [20].

### 2.4. DNA Damage by Exogenous Agents

Ionizing radiation consists of alpha, beta, gamma, neutron, and X-rays [23]:

Ionizing radiation of X-rays (used for medical treatment or radiotherapy, together with gamma irradiation and heavy beams): This type of radiation generates FRs from the water present in the cell, the most important being the hydroxyl radical (^•^OH). These radicals can induce damage in the 2′-deoxyribose moiety of DNA [24].

**Figure 8:**
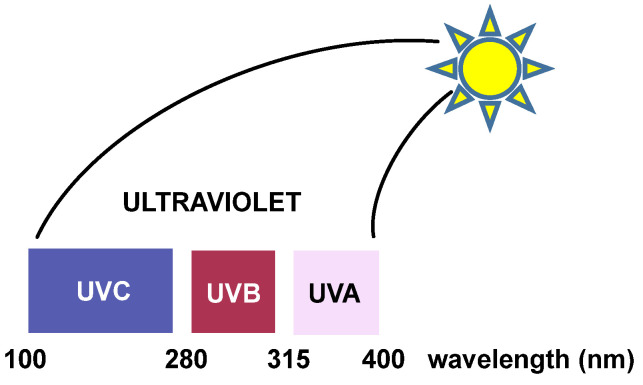
The three regions of UV radiation.

Ultraviolet (UV) radiation: Ultraviolet radiation is capable of causing mutations in various organisms, which are linked to photocarcinogenesis processes [25]. UV radiation is divided into three regions. “UVC” radiation covers wavelengths ranging from approximately 200 to 280 nm. This region of the spectrum does not reach the earth’s surface because it is filtered by the ozone layer present in the atmosphere [26]. Between 280 and 320 nm is the “UVB” region, which is largely filtered by the ozone layer in the atmosphere. UV light with longer wavelengths (“UVA” and “UVB”) significantly affects DNA and affects the purine and pyrimidine bases of nucleic acids [27] (Figure 8).

The excited states generated by direct absorption of radiation produce different types of reactions, the most common being those involving pyrimidinic bases. The pyrimidinic bases also undergo photochemical reactions when they absorb radiation, but to a lesser extent [28]. Ultraviolet radiation is the most damaging and mutagenic component of the solar radiation spectrum because DNA bases directly absorb the incident “UVB” photons. “UVA” light also exhibits cytotoxic and mutagenic characteristics, although to a lesser extent than “UVB” radiation.

The two DNA lesions mainly caused by “UVA” and “UVB” radiations are cyclobutene–pyrimidine dimers (CPD) and 6-4 photoproducts (PP). These cause structural distortions that ultimately result in replication stalls and double-strand breaks. The ozone layer absorbs the most dangerous part of the solar UV spectrum (“UVC”), but the residual “UVA” and “UVB” spectra can induce an average of 100,000 lesions per exposed cell in an hour [29].

UV radiation damage causes covalent bonding between two adjacent DNA pyrimidines (pyrimidine dimerization: thymine–thymine, thymine–cytosine, and cytosine–cytosine), which increases the probability of DNA polymerase inserting an incorrect nucleotide at the position with pyrimidine dimerization during DNA replication [30]. Pyrimidine dimerization can also occur between different DNA strands, which prevent the separation of the two DNA strands, blocking gene expression by stopping replication. In general, for any of the pyrimidine bases, dimerization reaches a maximum yield at 280 nm and monomerization is favored at 240 nm [31].

**Figure 9:**
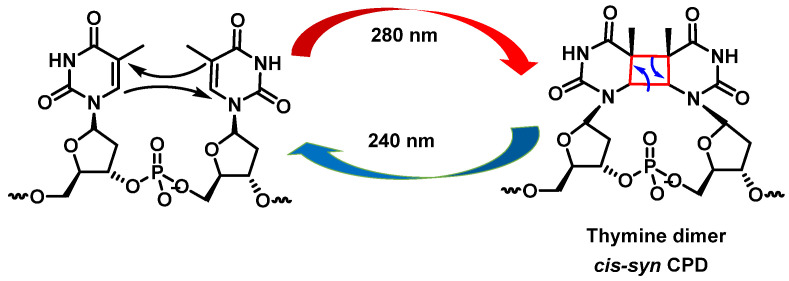
Formation of cyclobutanethymine dimer.

Cyclobutadipyrimidines are the main DNA photoproducts after exposure to “UVB” light [32]. They are formed by [2+2] cycloaddition of the C5-C6 double bonds of adjacent pyrimidine bases. Due to steric constraints, cis-synes is the major distereoisomer formed. The formation of the cyclobunatose derivative can be reversed by “UVC” irradiation [33] (Figure 9).

**Figure 10:**
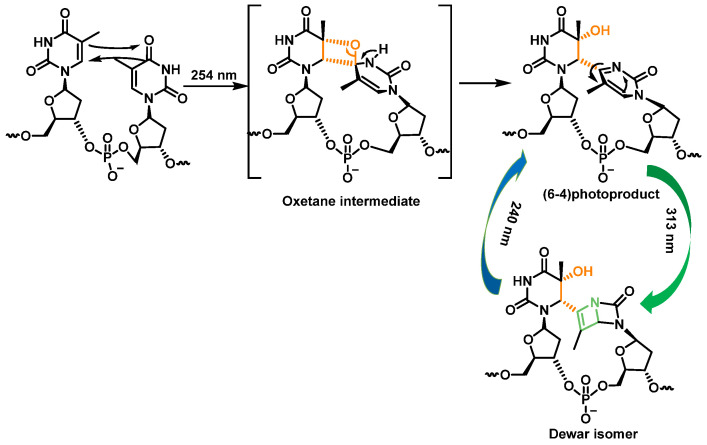
Formation and photoisomerization of thymine photoproduct (6-4).

Pyrimidine (6-4) pyrimidine adducts, whose formation involves a singlet excited state, represent the second class of pyrimidine photoproducts in terms of quantitative importance. They arise from a [2+2] cycloaddition involving the C5-C6 double bond of the 5′-end pyrimidine and the C4 carbonyl group of the 3′-end thymine. As a result, the formation of an oxetane, which is unstable, is obtained. An azetidine intermediate is generated when the 3′-end base is a cytosine, by cycloaddition of the 4-imino function of the last pyrimidine base. Spontaneous rearrangement of oxetane or azetidine produces pyrimidine (6-4) adducts [28]. CPD and (6-4) photoproduct comprise 70% to 80% and 20% to 30%, respectively, of the total photoproducts [34] (Figure 10).

## 3. Damage to DNA by ROS and RNS

DNA damage by endogenous and exogenous agents is a major problem, as the damaged products can severely affect the integrity of the genome. The study of FRs has become increasingly important in the fields of biology and medicine [35]. Overproduction of FRs can damage macromolecules, such as nucleic acids, lipids, and proteins [36]. This leads to tissue damage in various chronic and degenerative diseases. ROS and RNS include superoxide radical (O_2_^•−^), hydroperoxyl radical (HO_2_^•^), hydrogen peroxide (H_2_O_2_), hydroxyl radical (^•^OH), alkoxyl radical (RO^•^), peroxyradicals (RO_2_^•^), organic hydroperoxides (ROOH), nitric oxide (NO^•^), peroxynitrite (ONOO^−^), and singlet oxygen (^1^O_2_) [37].

**Figure 11:**
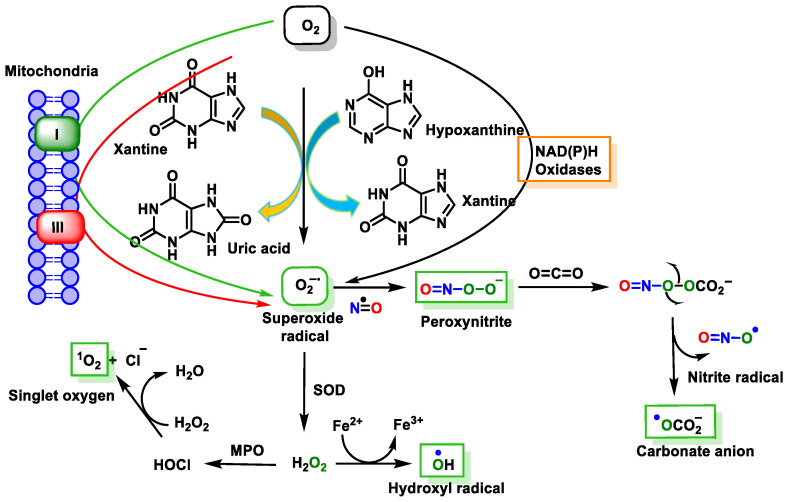
Source of free radical generation.

These reactive species can be produced by endogenous sources, such as cellular aerobic metabolism, and can also be produced by macrophages and neutrophils at sites of infection and inflammation as well as by exposure to various physical agents, such as UV light and ionizing radiation, or chemical pollutants or carcinogens [38] (Figure 11).

Endogenous or exogenous sources generate ROS (O_2_^•−^, ^•^OH, HO_2_^•^, H_2_O_2_, RO_2_^•^, and hypochlorous acid (HOCl)) and RNS (NO^•^, NO_2_^•^, ONOO^−^, nitrous acid (HNO_2_), alkylperoxynitrates (RONOO), and dinitrogen trioxide (N_2_O_3_). Endogenous FRs are produced by the activation of immune cells (eosinophils, neutrophils, etc.) to fight bacteria and other invaders [39] by the mitochondrial respiratory chain [40], by enzymatic activity (xanthine oxidase, NADPH oxidase, lipooxygenase, NO synthase, etc.) [41], and by various pathological disorders and diseases [42]. Exogenous FRs arise from air and water pollution, smoke, drugs, heavy or transition metals, industrial solvents, radiation, and high temperatures [42].

**Figure 12:**
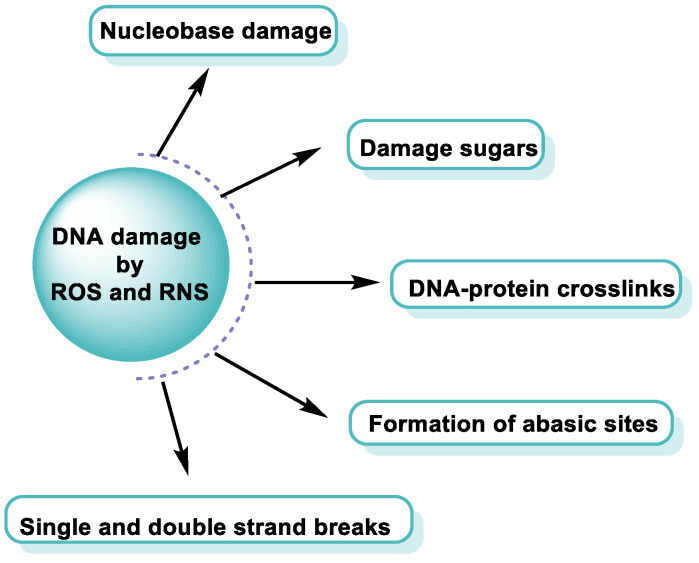
DNA damage by ROS and RNS.

ROS/RNS react with almost any molecule in cells and tissues, inducing changes in their biological function [43]. Exposure of DNA to these reactive species can result in damage, which must be repaired to avoid a transformation that can lead to lethal events, mutagenesis, malignant transformation, or genomic instability [44]. Structural integrity and biological functions of nucleic acids are altered by ROS/RNS, which can lead to cell apoptosis. Both the deoxyribose sugar and DNA nucleobases are susceptible to oxidative/nitrative attack by these reactive species [45] (Figure 12).

**Figure 13:**
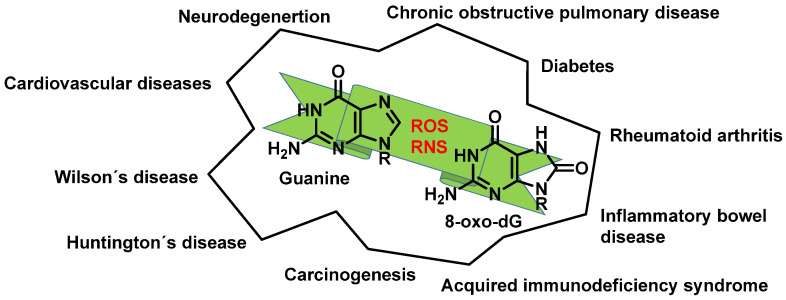
Diseases resulting from ROS and RNS reacting with guanine.

Any of these processes can have serious consequences and have been implicated in various pathological conditions (Figure 13).

## 4. Major Damage to the DNA Structure

### Modifications of Nitrogenous Bases

**Figure 14:**
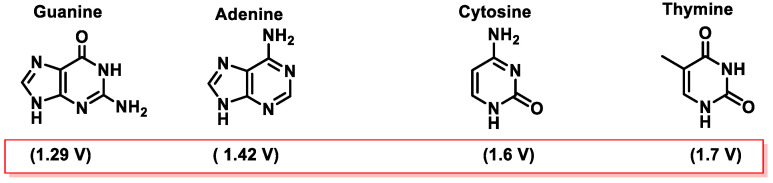
Values of the standard reduction potentials of DNA bases.

Among the various pathways of DNA damage, base modification is the most common. DNA base modification generally refers to changes in the structures and chemical properties of the bases, so that their functionality will be affected. Gua, Ade, Cyt, and Thy can receive or donate electrons depending on their chemical environment. The tendency of a molecule to be an electron donor or acceptor is determined by calculating its oxidation–reduction (redox) potential. In the case of the nitrogenous bases that make up the DNA, the redox potentials of nucleosides have been published. The redox potentials of deoxynucleosides determined experimentally at pH = 7 are as follows: deoxyguanosine = 1.29 V, deoxyadenosine = 1.42 V, deoxycytidine = 1.6 V, and deoxythymidine = 1.7 V [46]. From these data, data for nucleobases have been extrapolated [47,48] (Figure 14).

Guanine is the nitrogenous base with the lowest standard reduction potential among all DNA bases and is therefore the most susceptible to oxidation by different oxidants, such as the ^•^OH, ^1^O_2_, O_2_^•−^, ONOO^−^, and CO_3_^•−^ radicals [49,50] (Figure 14).

**Figure 15:**
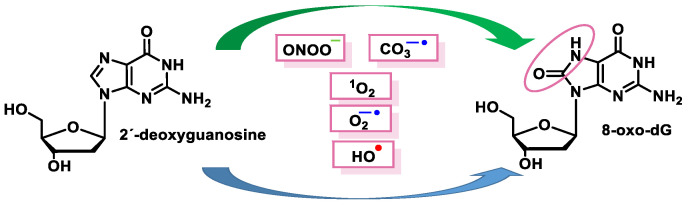
Schematic summary of the 8-oxo-dG generation.

These radicals can chemically modify the nitrogenous bases, leading to mispairing. The oxidation product of the C8 oxidation of the imidazole ring of deoxyguanosine (dG) generates 8-oxo-dG (8-oxoguanosine, tautomer known as 8-hydroxyguanosine). 8-oxo-dG can be formed at the level of free nucleotides (8-oxo-GTP) or directly at the level of the DNA molecule and represents the most stable and common product of DNA oxidation [51] (Figure 15).

Among the more than one hundred possible oxidative lesions that can occur in DNA, 8-oxo-dG is undoubtedly the most studied and documented [52]. However, there exist additional important oxidation products involving DNA nucleobases, such as 2,6-diamino-4-diamino-4-hydroxy-5-formamidopyrimidine (also resulting from guanine oxidation), 7,8-dihydro-8-oxo-adenine, 4,6-diamino-5-formamidopyrimidine, and ethenoadenine (resulting from the oxidation of adenine), and thymine glycol, 5-hydroxycytosine, and dihydrouracil (all resulting from the degradation of the pyrimidine nitrogenous bases) [53,54].

^1^O_2_ was shown to be the main reactive species responsible for the formation of 8-oxo-dG in DNA, as it appears to be reacting exclusively with guanine and not with the other bases [55]. The proposed mechanism involves the formation of an endoperoxide intermediate, through the addition of oxygen to the five-membered ring of guanine, similar to Diels–Alder (4+2) cycloaddition reaction. The unstable species undergoes rearrangement, with the formation of an intermediate peroxide species, which is eventually reduced to 8-oxo-dG [56].

Once formed, 8-oxo-7,8-dihydroguanine (8-oxoG) undergoes several degradation reactions that produce various mutagenic species. The various products formed by reactions of guanine and 8-oxoG with different reactive species are mainly 2,6-diamino-4-oxo-5-formamidopyrimidine, 2,5-diamino-4H-imidazolone, 2,2,4-triamino-5-(2H)-oxazolone, 5-guanidino-4-nitroimidazole, guanidinohydantoin, spiroiminodihydantoin, cyanuric acid, parabanic acid, oxaluric acid, urea, among others [57].

**Figure 16:**
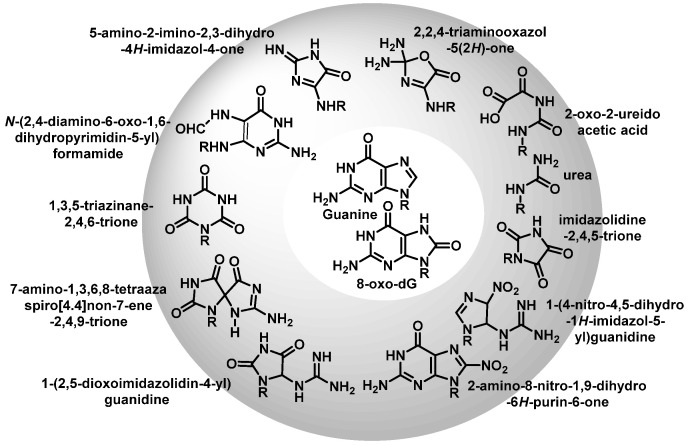
Various products formed by reactions of G or 8-oxoG.

These products are formed from: (i) ring opening and (ii) ring opening and subsequent rearrangement. Nucleobase modifications are possible on both the nucleobase and the nucleobase radical cation (Figure 16).

**Figure 17:**
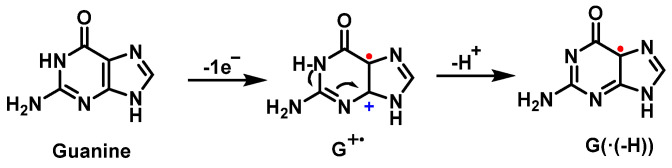
Guanine oxidation mechanism.

The first step of the guanine oxidation mechanism is the loss of an electron, which can be induced by ROS such as the ^•^OH or the carbonate radical [58,59]. In this first step, a cation radical (G^+^) is formed, whose relatively high acidity (pKa = 3.9) causes it to deprotonate in physiological media, producing the species G(−H)^•^, a neutral guanine radical [60] (Figure 17).

**Figure 18:**
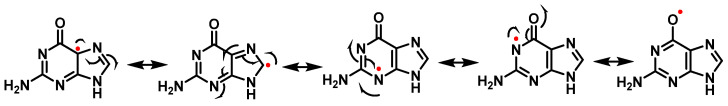
Resonant forms of the guanine radical.

The odd electron of such a radical is delocalized and has various resonant forms (Figure 18). Many mechanisms involve the addition of an oxidizing agent to the radical.

The reaction of G(−H)^•^ with radicals such as O_2_^•−^, NO_2_^•^, and CO_3_^•−^ occurs rapidly and results in the formation of DNA lesions by oxidation. A major product of this process is Iz, and its hydrolysis product is Z [61].

## 5. Superoxide Anion and DNA Damage

**Figure 19:**
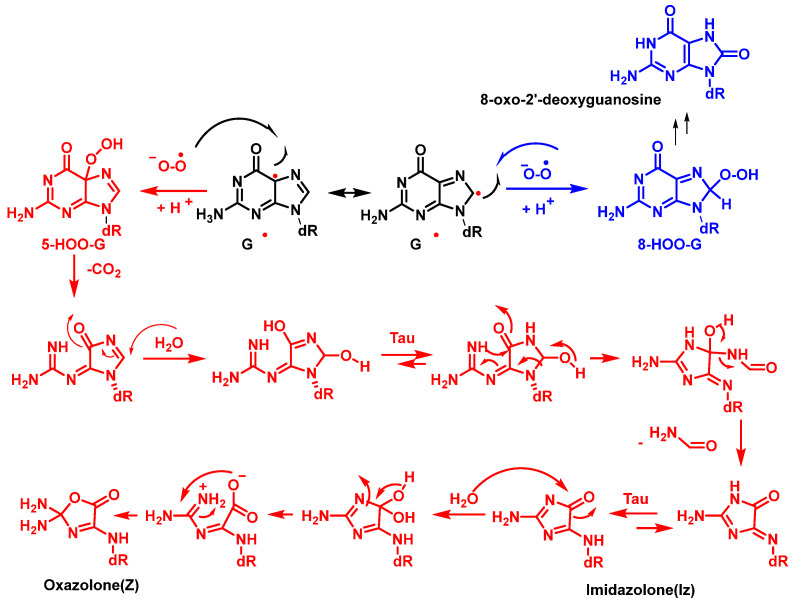
Formation of imidazolone, oxazolone adducts, and 8-oxodG via the combination of G and O_2_^•−^ radicals in DNA. dR is deoxyribose.

The superoxide radical ion is a precursor of many ROS [62]. It is generated by incomplete reduction of molecular oxygen during electron transport in the mitochondria and endoplasmic reticulum. O_2_^•−^ is also produced by macrophages and neutrophils as part of the immune defense system by NADPH-mediated reduction of O_2_. Other sources of superoxide radicals include enzymatic reactions, such as those carried out by xanthine oxidase and by metabolic activation of xenobiotics. O_2_^•−^ is a short-lived radical species that can act as a reducing agent at neutral pH and as an oxidant at pHs lower than its pKa (4.8) [63]. Due to its anionic nature, superoxide does not freely cross biological membranes [64]but the neutral perhydroxyl radical does (HO_2_^•^) [65]. Cadet et al. reported that a O_2_^•−^ does not oxidize DNA because it is relatively unreactive with DNA [66]. Among the nucleobases, guanine is most readily oxidized, but has no reactivity with O_2_^•−^ [67]. However, the guanine radical, formed at C-5 and C-8, can be oxidized by O_2_^•−^, forming 5- and 8-hydroperoxides, which subsequently evolve into the oxidized products, imidazolone and oxazolone and 8-oxo guanine derivatives, respectively. According to the mechanism proposed by Cadet et al. [59], cleavage of 5-HOO-G(-H) occurs via pyrimidine ring opening at the C-5-C-6 bond in 5-HOO-G(-H), leading to an unstable intermediate that readily hydrates at the 7,8-C=N double bond. Tautomerization of the carbinolamine ring chain results in imidazole ring opening with subsequent intramolecular cyclisation of the guanidine residue, resulting in the imidazolone lesion (Figure 19).

## 6. Singlet Oxygen and DNA Damage

^1^O_2_ reacts with the DNA molecule, preferably with guanine to form 8-oxodGuo, and from this reaction, multiple products can be formed [66]. In principle, all four bases are susceptible to oxidative damage by ^1^O_2_. However, due to the low reduction potential of guanine, it is more susceptible to oxidation [46]. 7,8-dihydro-8-oxo-2′-deoxyguanosine (8-oxodG) has been shown to be the major product of the reaction of ^1^O_2_ with free deoxynucleosides [68,69,70,71]. However, Niles et al. identified different diastereoisomers of spiroiminodihydantoin as the main end product formed during methylene blue-mediated photo-oxidation of guanosine [72].

Of the four DNA nucleobases, ^1^O_2_oxidizes only guanine, which is the most oxidizable of the nucleobases [73]. In addition, ^1^O_2_ induces strand breaks in DNA; however, it is much less frequent than the oxidation of guanine to 8-oxo-guanine [74].

**Figure 20:**
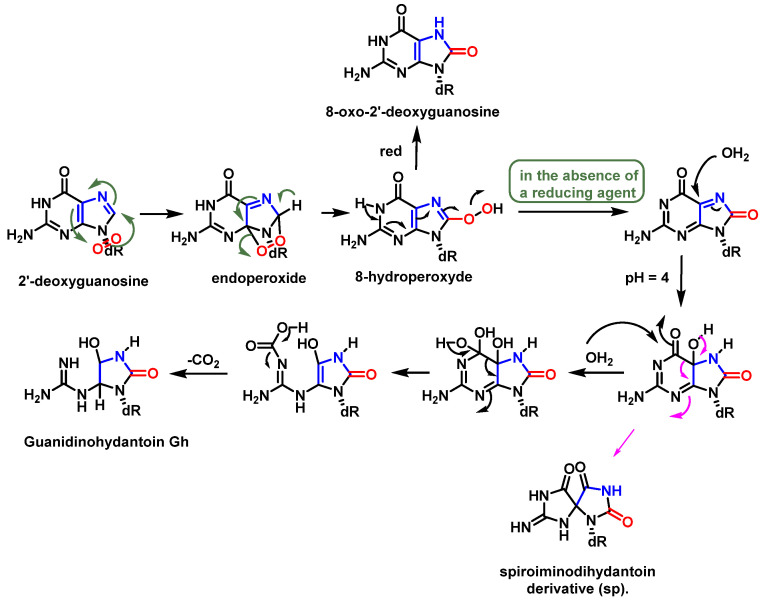
Mechanism of formation of spiroiminodihydantoin (Sp).

Guanosine (Gua) is the only DNA component that reacts significantly with ^1^O_2_ at neutral pH [66]. The oxidation of Gua by ^1^O_2_ is believed to involve the formation of an unstable endoperoxide as a result of a Diels–Alder (4+2) cycloaddition reaction [75]. Initially, the two diastereoisomers (4R* and 4S*) of 4-hydroxy-8-oxo-4,8-dihydro 2′-deoxyguanosine (8-oxodG) were considered as the stable products of endoperoxide decomposition [76]. Subsequently, it was shown that the diastereoisomers rearrange to produce spiroiminodihydantoin(Sp) [77] (Figure 20).

## 7. Hydroxyl Radical and DNA Damage

The ^•^OH radical reacts directly with all DNA components, such as the purine and pyrimidine bases and the deoxyribose sugar backbone, causing alterations, including single- and double-strand breaks. It abstracts hydrogen atoms, modifying purines and leading to pyrimidine derivatives and DNA–protein cross-linking. ^•^OH is responsible for the most frequent oxidative damage to DNA [78,79], as it is the most reactive and electrophilicROS [80]. It reacts with most biomolecules at diffusion-controlled rates (k > 10^9^ M^−1^s^−1^). Due to its high reactivity, its half-life is extremely short (10^−9^ s) [81].

^•^OH radicals react with all purine and pyrimidine bases, as well as with the deoxyribose backbone, generating base and sugar by-products [82]. In addition, ^•^OH reacts with proteins surrounding DNA (e.g., histones) and can produce DNA–protein cross-links [83].

**Figure 21:**
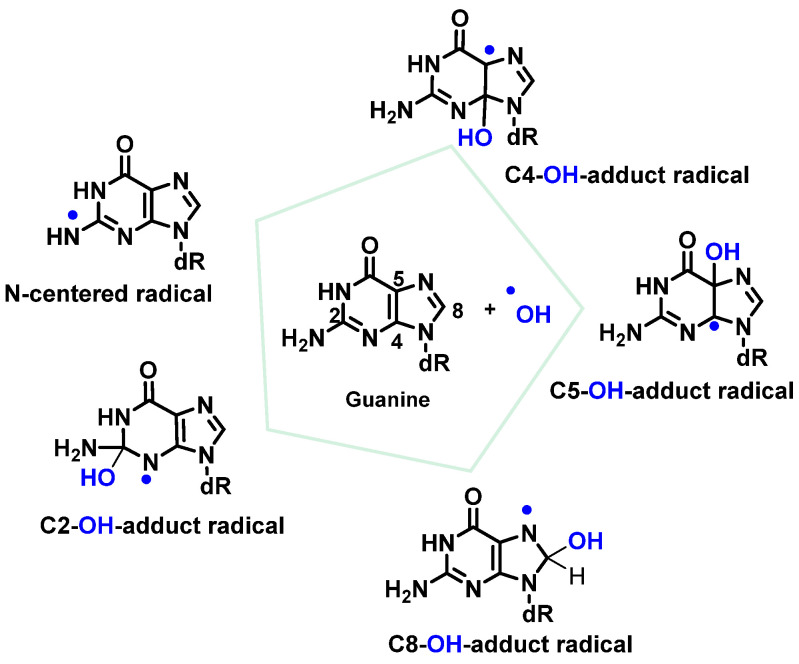
Reactions of ^•^OH with Gua.

^•^OH radicals react mainly with heterocyclic bases, resulting in heterocyclic-derived radicals that are irreversibly transformed. The products of the oxidation of thymine, cytosine, and guanine by ^•^OH radicals are described below [82]. ^•^OH attacks are preferentially focused on guanine residues and generate radical adducts, and ^•^OH is added to the C4, C5, and C8positionsof guanine, and to the C2 position to a much lesser extent [84,85,86]. An abstraction of an H^•^ from NH_2_ adjoining C2 by ^•^OH can also occur. Due to the electrophilic nature of ^•^OH, additions occur preferentially at the electron-dense G-positions, providing OH-adduct radicals [78,87,88,89] (Figure 21).

**Figure 22:**
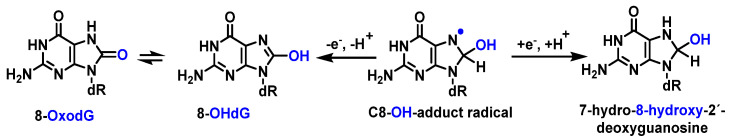
Mechanisms of formation of 8-oxoG by addition of OH to guanine at C8 in DNA.

The C8-OH-adduct radical produces the main product of Gua in DNA. The abstraction of an electron forms, among other derivatives, 8-hydroxy-2′-dxyguanosine (8-OHdG). 8-OHdG undergoes keto–enol tautomerization, leading to the oxidized product 8-oxodG [90] (Figure 22).

Guanine has the lowest redox potential [91]; therefore, it forms 8-oxo-dG, which is the oxidized form generated by reaction of ^•^OH with the C8 position of guanine. It is estimated that 8-oxo-dG is present in approximately 0.5 per Mbp (million base pairs; steady state of the human lymphocyte genome) [92].

**Figure 23:**
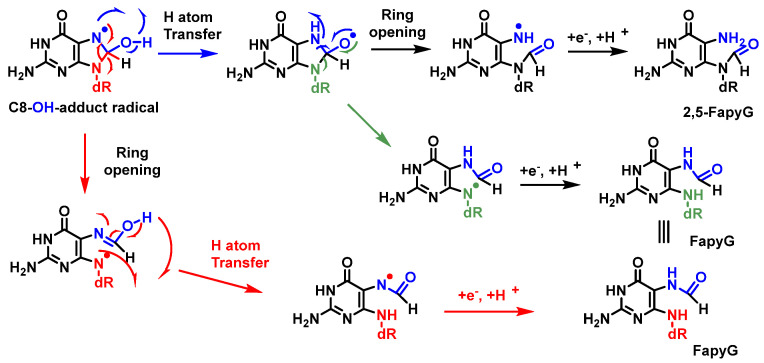
Mechanisms of formation of FapyG and 2,5-FapyG from C8-OH-radical adduct.

A competitive reaction of the 8-hydroxy-7,8-dihydroguanyl radical (8-HO-G) involves the opening of the imidazole ring by breaking the C8-N9 bond of the guanine derivative, followed by the reduction of an electron, leading to the formation of 2,6-diamino-4-hydroxy-5-formamidopyrimidine (FapyG) and the possible formation of the 2,5-FapyG isomer, under reductive conditions (Figure 23). The 2,5-FAPyG adduct is thermodynamically less stable than the FAPyG adduct [93].

Formamidopyrimidines, i.e., purine derivatives such as 4,6-diamino-5-formamidopyrimidine (FapyAde), and 2,6-diamino- 4-hydroxy-5-formamidopyrimidine (FapyG), are among the major DNA lesions generated by ^•^OH attack, UV radiation, or photosensitization under numerous in vitro and in vivo conditions. They are formed from the C8-OH-adduct radicals of purines. Methodologies using mass spectrometry are available to accurately measure the amount of FapyAde and FapyG in vitro and in vivo [94].

As with guanine, the ^•^OH reacts with adenine by addition to its double bonds, but the distribution of the additions is somewhat different. Thus, additions at C4 and C8 amount to 50% and 37%, respectively, forming C4-OH- and C8-OH-adducts, and the addition of ^•^OH to C5 produces the C5-OH-radical adduct and is 5% of the products formed, whereas its addition at C2 is about 2%, due to the low electron density at this position [85,95,96].

**Figure 24:**
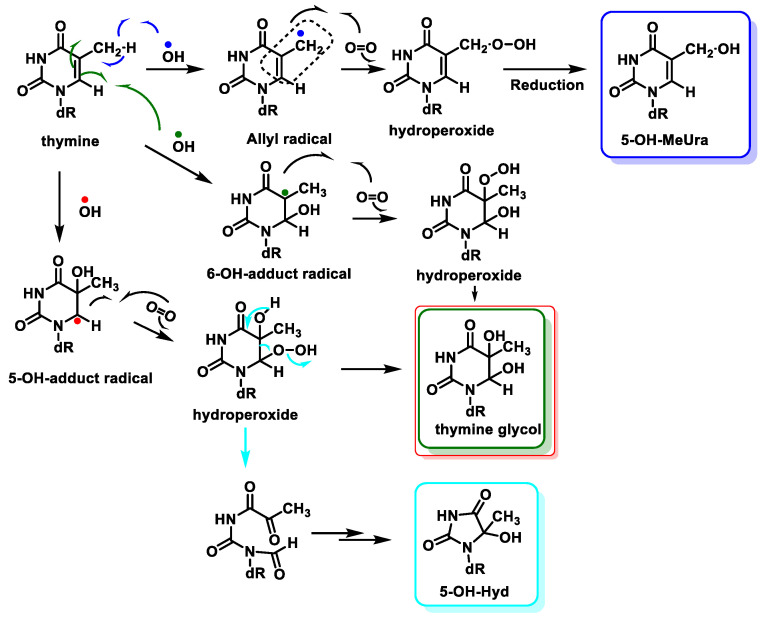
Reactions of ^•^OH with thymine and mechanisms of product formation from reactions of the C5-OH– and C6-OH–adduct radicals and the allyl radical of thymine with O_2_.

The main site for the reaction of the ^•^OH with the pyrimidine bases is the 5,6 double bond. ^•^OH adds to the C5-C6 double bonds of Thy, with up to 60% at C5 and 30% at C6, and also abstracts an H^•^ from the methyl group to a much lesser extent (10%) [97,98]. Thymine is transformed into hydroperoxides, which can be reduced to thymine glycols or degraded to 5-hydroxyhydantoin (5-OH-Hyd) via the open-chain intermediate. Thymine can also undergo hydrogen abstraction from methyl, generating an allylic radical, which is produced by the reaction of thymine with oxygen and a subsequent reduction of the hydroperoxide to 5-(hydroxymethyl)uracil (5-OH-MeUra) (Figure 24). Thymidine glycol exhibits a high mutagenic capacity towards DNA molecules, causing lethal oxidative damage [99].

**Figure 25:**
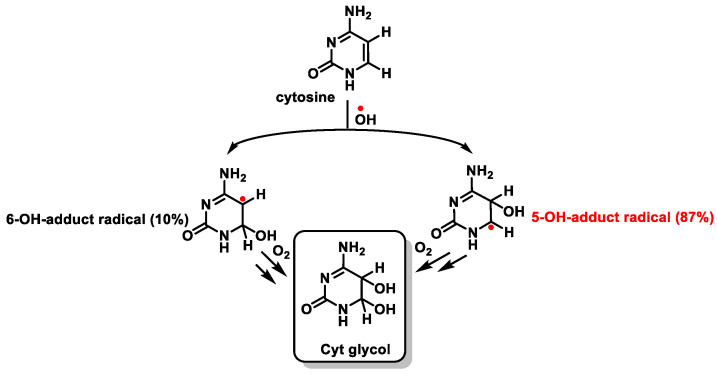
Reaction of cytosine with ^•^OH and formation of Cyt glycol.

The behavior of Cyt towards ^•^OH is similar to that described for thymine. The addition of the ^•^OH to the C5-C6 double bond generates the C5-OH- (87%) and C6-OH-adduct (10%) radicals, which form Cyt glycol [100] (Figure 25).

This distribution is the important difference with respect to the addition of ^•^OH to Thy and is due to the higher electron density at C5 compared to that at C6 [101,102]. The C5-OH^•^ radical has reducing properties, while the C6-OH^•^ adduct radical is a weak oxidant.

**Figure 26:**
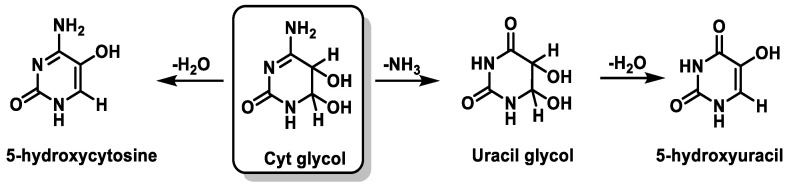
Deamination and dehydration of the Cytglycol.

Cyt derivatives are unique in that they can undergo dehydration and deamination. Thus, Cyt glycol produces 5-hydroxycytosine by dehydration, uracil glycol by deamination, and 5-hydroxyuracil by deamination followed by dehydration [103,104] (Figure 26).

Currently, more than 100 oxidative DNA products have been identified, ranging from those with base modifications (e.g., 8-oxo-dG, 8-oxo-dA, thymidine glycol, 5-hydroxycytosine, and 5-hydroxyuracil) and nucleotides (abasic or cyclic forms, e.g., 2′-deoxyribonolactone, 5′,8-cyclo-2′-deoxyguanosine, and 5′,8-cyclo-2′-deoxyadenosine) to those with a cleaved phosphate skeleton (e.g., 5′,8-cyclo-2′-deoxyadenosine) [105].

## 8. Peroxynitrite and DNA Damage

**Figure 27:**
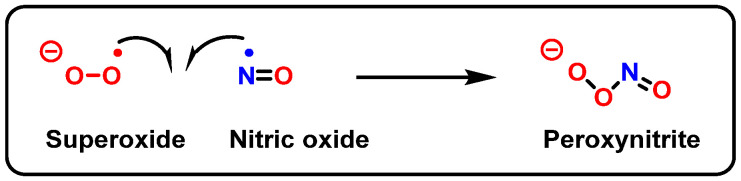
Formation of peroxynitrite.

Peroxynitrite is formed in biological systems when nitric oxide and superoxide interact rapidly in an almost equimolar ratio (10^9^ M^−1^ s^−1^) (Figure 27). However, because NO^•^ is diluted by diffusion and the presence of SODs, only a small part (1%) of the O_2_^•−^ produced reacts with NO^•^ to form ONOO^−^. Most of the O_2_^•−^ is converted to H_2_O_2_ by SOD. Peroxynitrite is not a free radical by its chemical nature, but it is a powerful oxidant and nitrating agent with a short half-life (~10 ms), which reacts with DNA [106,107].

**Figure 28:**
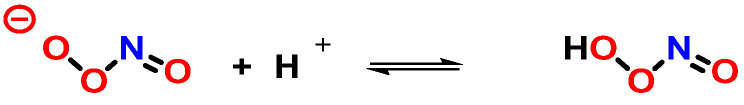
Protonation of peroxynitrite leads to the formation of peroxynitrous acid, ONOOH.

At neutral pH, ONOO^−^ coexists in equilibrium with the unstable ONOOH, and their rapid protonation means that, under most biological conditions, both ONOO^−^ and ONOOH will be present [108] (Figure 28).

**Figure 29:**
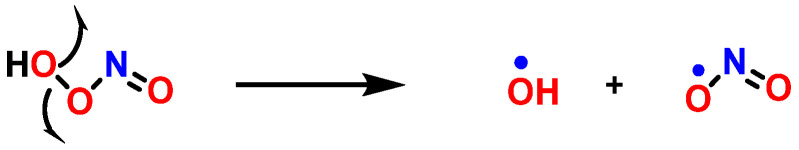
Decomposition of peroxynitrite.

Protonation weakens the O-O bond in ONOOH and leads to homolytic cleavage to form^•^OH and ^•^NO_2_, two strong oxidizing/hydroxylating and nitrating species, respectively [109] (Figure 29).

**Figure 30:**
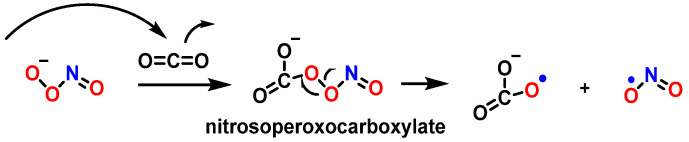
Homolysis of nitrosoperoxocarboxylate.

As a nucleophile, an important reaction of peroxynitrite in biology is the addition of the anion to carbon dioxide to produce a nitrosoperoxocarboxylate adduct (ONOOCO_2_^−^), which undergoes rapid homolysis to ^•^NO_2_ and CO_3_^•−^ (Figure 30) [110].

**Figure 31:**
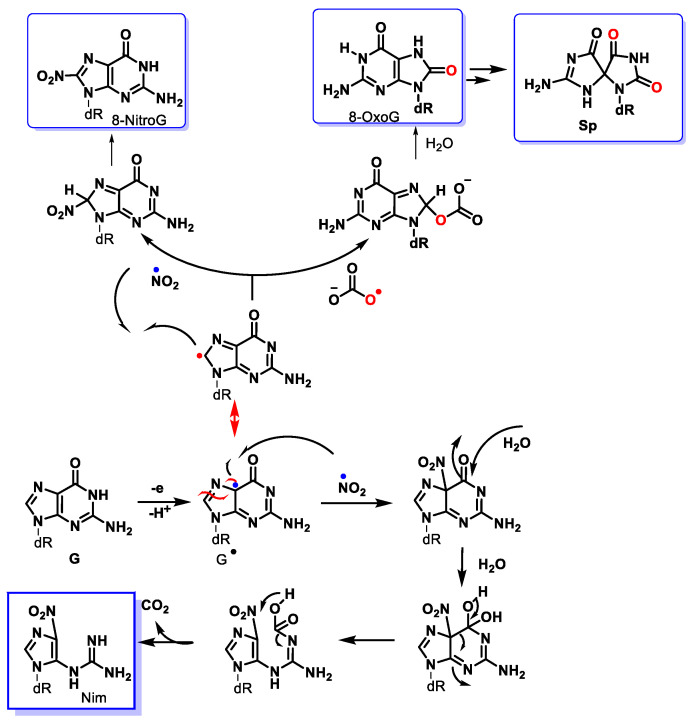
Mechanism of formation of main products from the reaction of peroxynitrite with the guanine derivative.

ONOO^−^ diffuses readily across cell membranes [39] and can interact with guanine to produce nitrative and oxidative lesions in DNA [111]. The high levels of bicarbonate in interstitial (30 mM) and intracellular fluids (12 mM) suggest that the reaction between ONOO^−^ and CO_2_ is the main pathway for peroxynitrite breakdown in biological systems. The formation of 8-nitroguanine (8-nitroG) occurs by the addition of ^•^NO_2_ to the C8 position. Attack of ^•^NO_2_ at its C5 position generates unstable adducts, which rapidly decompose to 5-guanidino-4-nitroimidazole (NIm) [112,113] (Figure 31).

In DNA, purine nucleotides are susceptible to oxidation and adduct formation with 8-oxo and 8-nitroguanine existing as two of the main products [114,115,116,117]. DNA damage caused by peroxynitrite is mainly oxidative.

**Figure 32:**
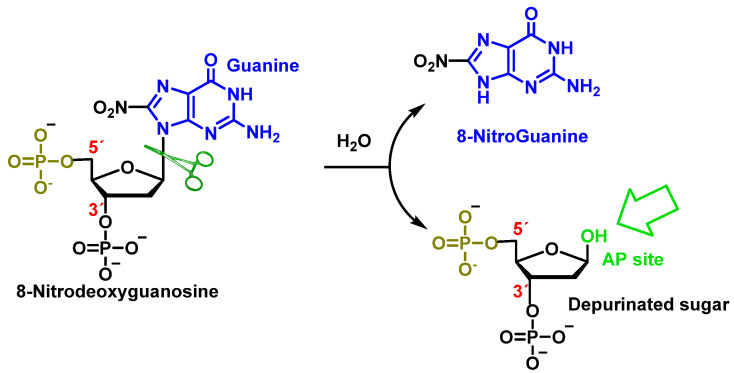
Formation of 8-nitroguanine by depurination of 8-nitro-2′-deoxyguanosine generated with peroxynitrite.

Peroxynitrite can also cause deoxyribose oxidation and strand breaks [118]. The resulting 8-nitroguanine is unstable and can be spontaneously eliminated, resulting in an apurinic site (Figure 32). Conversely, adenine can couple with 8-nitroguanine during DNA synthesis, resulting in GT transversions. Therefore, 8-nitroguanine is a DNA mutagenic lesion involved in carcinogenesis [119].

## 9. Carbonate Radical Anion and DNA Damage

**Figure 33:**
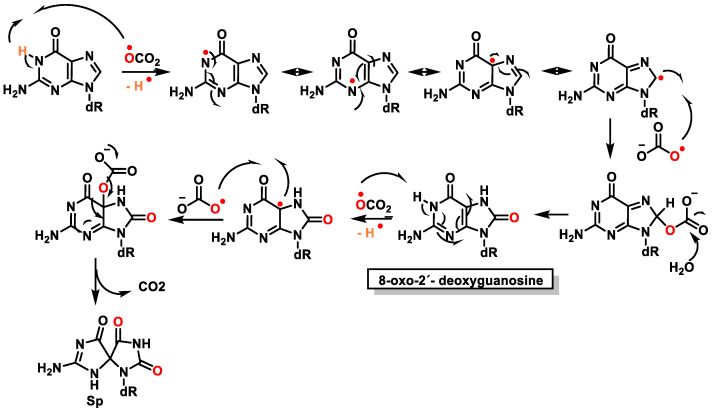
Mechanism of formation of 8-Oxo-dG and Spby carbonate radical attack.

The carbonate radical anion is formed from the Fenton reaction under cellular conditions and from the decomposition of nitrosoperoxycarbonate generated during inflammation [120]. The carbonate radical anion (CO_3_^•−^) can be produced by (i) the radiolysis of aqueous bicarbonate/carbonate solutions [121] and(ii) when ^•^OH reacts with carbonate or bicarbonate ions. Bicarbonate levels are high (25 mM) in blood plasma, which allows the reaction to occur [122]. Although not as strong an oxidizing agent as the hydroxyl radical, the CO_3_^•−^ is a strong one-electron oxidant that acts by electron transfer and hydrogen removal [39]. It has a much longer half-life than ^•^OH and can therefore spread further and oxidatively modify distant cellular targets, and CO_3_^•−^ can oxidize a wide variety of biomolecules [123]. Although it has a lower standard reduction potential than ^•^OH (E_OH/H2O_ = 2.3 V), CO_3_^•−^ is a strong oxidant (E_CO3_^•−^_/HCO3_^−^= 1.7 V). In vitro studies have shown that CO_3_^•−^ rapidly and more specifically oxidizes guanine residues in DNA [124]. Most reactions of this radical are oxidations occurring in aqueous media by electron transfer and hydrogen abstraction, but it almost never produces addition reactions. Considered a major oxidant of proteins and nucleic acids, it oxidizes the guanine bases of DNA by a one-electron transfer process, leading to the formation of stable guanine oxidation products [125] (Figure 33).

CO_3_^•−^ is a more stable radical than ^•^OH. The diffusion of CO_3_^•−^ to other cellular compartments is limited because it is a charged species as is the case ofO_2_^•−^, and for this reason, its reactivity will be limited to the compartment in which it is generated [126].

**Figure 34:**
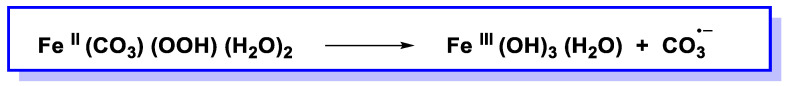
Reaction of metal-bound carbonate and hydroperoxide generates carbonate radical anion.

Fleming et al., in recent studies [127,128,129], have suggested that ^•^OH is not involved in DNA damage caused by oxidative stress and argue that CO_3_^•−^plays a key role in attacking guanine residues in DNA to form 8OHdG, causing in vivo oxidative damage to DNA. However, Halliwell’s group examines these claims and concludes that ^•^OH plays a key role and is an important member of reactive oxygen species (ROS) in vivo [130]. HCO_3_¯ is important in maintaining physiological pH and, in fact, is present intracellularly at a concentration between 10 and 40 mM. In vitro studies have suggested that, in the presence of HCO_3_¯, the reaction of Fe^2+^ and H_2_O_2_ does not generate ^•^OH but CO_3_^•−^ [120] (Figure 34).

**Figure 35:**
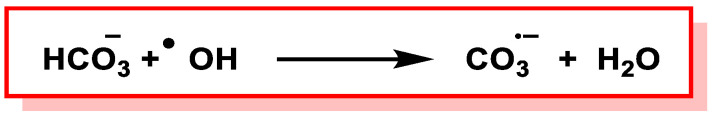
Formation of CO_3_^•^¯via H-atom abstraction from HCO_3_¯ by ^•^OH.

An alternative explanation is that ^•^OH is generated but reacts immediately with HCO_3_¯ to produce CO_3_^•−^. However, the rate constant for the formation of CO_3_^•−^ by the abstraction of H atoms from HCO_3_¯ by ^•^OH under physiological conditions has been measured using pulse radiolysis and has been found to be quite low, i.e., 8,5 × 10^6^ M^−1^ s ^−1^ [131], (Figure 35).

Molecules, such as 2′-deoxyribose phosphate, the pyrimidine and pyrimidine bases of DNA and RNA, reduced glutathione (GSH), and proteins, present in vivo, react much faster with ^•^OH, at diffusion-controlled rates (>10^9^ M^−1^ s^−1^), and may therefore be preferred targets, depending on the location and the environment in which the ^•^OH is generated. A combination experiment of laser flash photolysis and analysis studies of formed products has confirmed that CO_3_^•−^ oxidizes guanine in DNA to form 8OHdG [112,132], but there is no literature evidence of adenine oxidation by CO_3_^•−^.

According to the values of the absolute reduction potentials (E°), the direct experimental results show that, when ^•^OH reacts with DNA, it forms a multiplicity of damage products of the four purine and pyrimidine bases. No other known ROS forms such a wide range of products. H_2_O_2_ and O_2_^•−^ do not react directly with DNA, whereas others (e.g., CO_3_^•−^ and ^1^O_2_) selectively target guanine [127,133,134].

## 10. Damage to the Sugar of DNA

Damage to 2′-deoxyribose in DNA results in different products such as strand breaks and abasic sites, and consequently, in the release of unaltered DNA bases [135]. When C-centered radicals are produced, they undergo further reactions, producing a variety of 2′-deoxyribose-derived products. Some products are released from the DNA, while others remain within the DNA or constitute end-groups of broken DNA strands [136].

**Figure 36:**
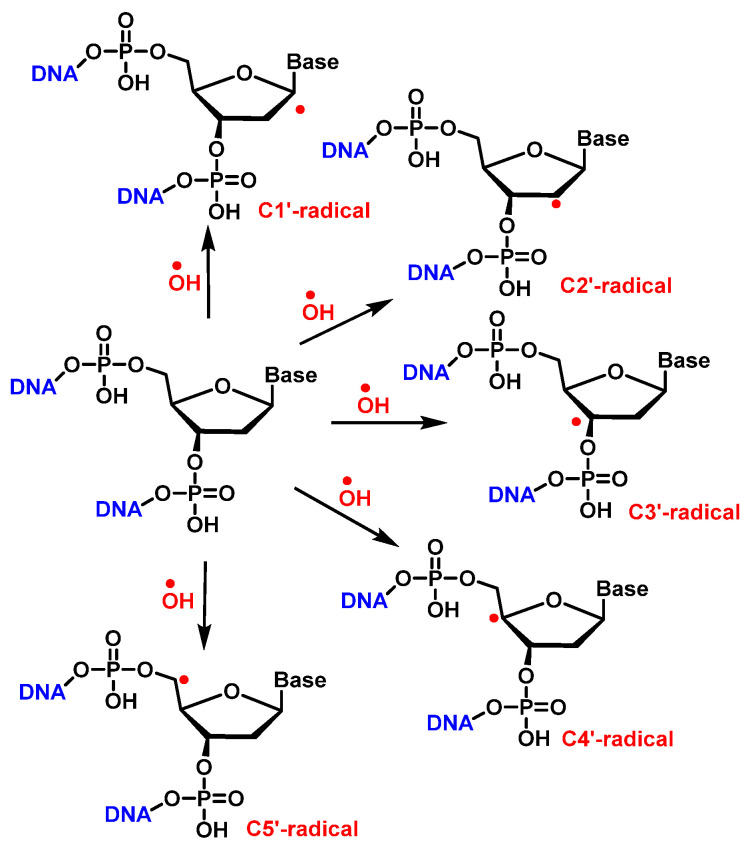
H^•^ abstraction by ^•^OH from 2′-deoxyribose in DNA.

^•^OH reacts with the 2′-deoxyribose in DNA by H-atom abstraction of all its carbons, leading to five radicals centered on C [137], as shown in Figure 34. The extent of -OH attack on 2′-deoxyribose in DNA typically amounts to less than 20% [130]. H4′ and H5′ are more accessible for H-atom abstraction by -OH than the other H atoms. The accessibility of H1′ is very low in the case of the two-stranded B form of DNA. The C4′ radical appears to be the main radical produced by H-atom abstraction of 2 ′-deoxyribose in DNA [138]. However, both experimental results and calculations showed that radicals centered on C4′ and C5′ cause strand breaks to approximately the same extent [83,139] (Figure 36).

**Figure 37:**
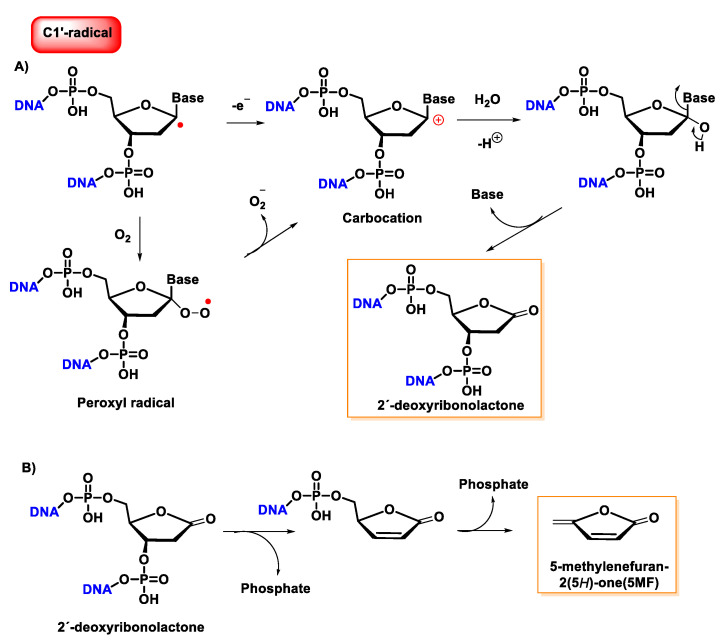
(**A**) Mechanism of formation of 2′-deoxyribonolactone from the oxidation of the C1′radical of 2′-deoxyribose and by reaction with oxygen. (**B**) Mechanism of formation of 5-methylenefuran-2-one (5MF) from 2′-deoxyribonolactone.

Once formed, the radical at C1′ can be further oxidized to form a carbocation, while in the presence of molecular oxygen, a peroxyl radical is formed, followed by the release of a superoxide anion and the formation of a carbocation at the C1′ position. The carbocation formed by either of the above two pathways is hydrolyzed by water; this process is accompanied by the release of the base and the formation of 2′-deoxyribonolactone [140], which is unstable and undergoes β-elimination of 3′-phosphate to provide a butenolide that has a half-life of 20 h in single-stranded DNA (32–54 h in duplex DNA), followed by rapid elimination and δ-elimination of 5′-phosphate on heating or at a basic pH to form 5-methylenfuran-2-one (5MF) [141,142,143] (Figure 37).

Several research groups have concluded that 2-deoxyribonolactone is potentially a highly toxic product that damages DNA [144,145]. 

**Figure 38:**
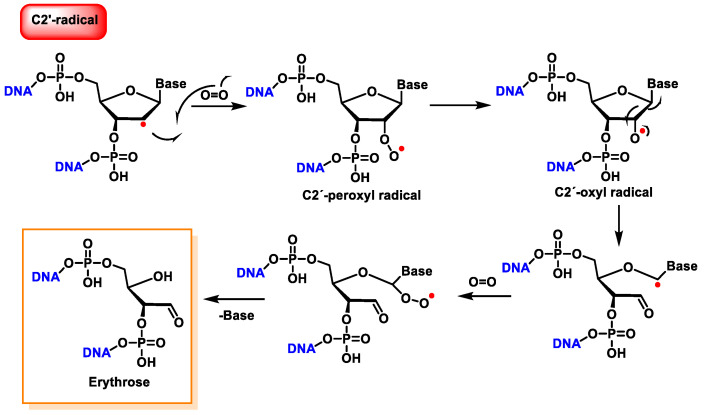
Reaction of the C2′ radical of 2′-deoxyribose with O_2_, leading to erythrose formation within DNA.

The reaction of the C2′ radical of the 2′-deoxyribose in DNA with O_2_ generates a peroxyl radical, which is subsequently transformed into an oxyl radical. The β-fragmentation of the latter radical, followed by the reaction with O_2_ and the release of the base, produces erythrose within DNA (Figure 38) [146].

**Figure 39:**
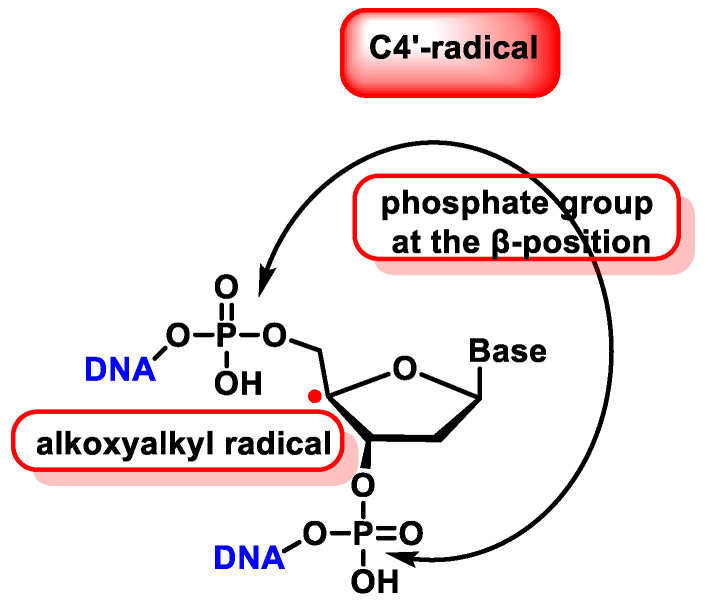
Properties of C4′ radical derived from 2′-deoxyribose.

The C4′ radical is an alkoxyalkyl radical with two phosphate groups in the β-position (Figure 39).

**Figure 40:**
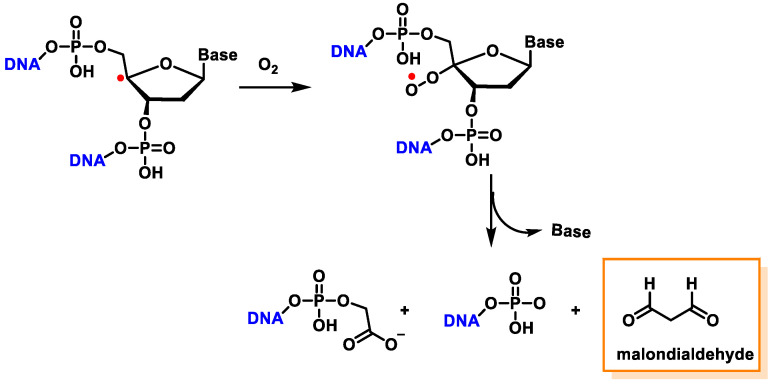
The formation of 3′-phosphoglycolate, 5′-phosphate, and MDA with the C4′ chemistry.

The C4′ pathway is initiated by the removal of hydrogen from the C4′ position of 2′-deoxyribose of DNA. This occurs majorly because of the accessibility of this site in B-DNA, and thus, many DNA-cleaving molecules can attack DNA at this position. In oxygenated solutions, several products are generated via the C4′ radical pathway, including 5′-phosphate, 3′-phosphoglycolate, the free and unaltered base, and malondialdehyde [139]. These products are shown in Figure 40.

**Figure 41:**
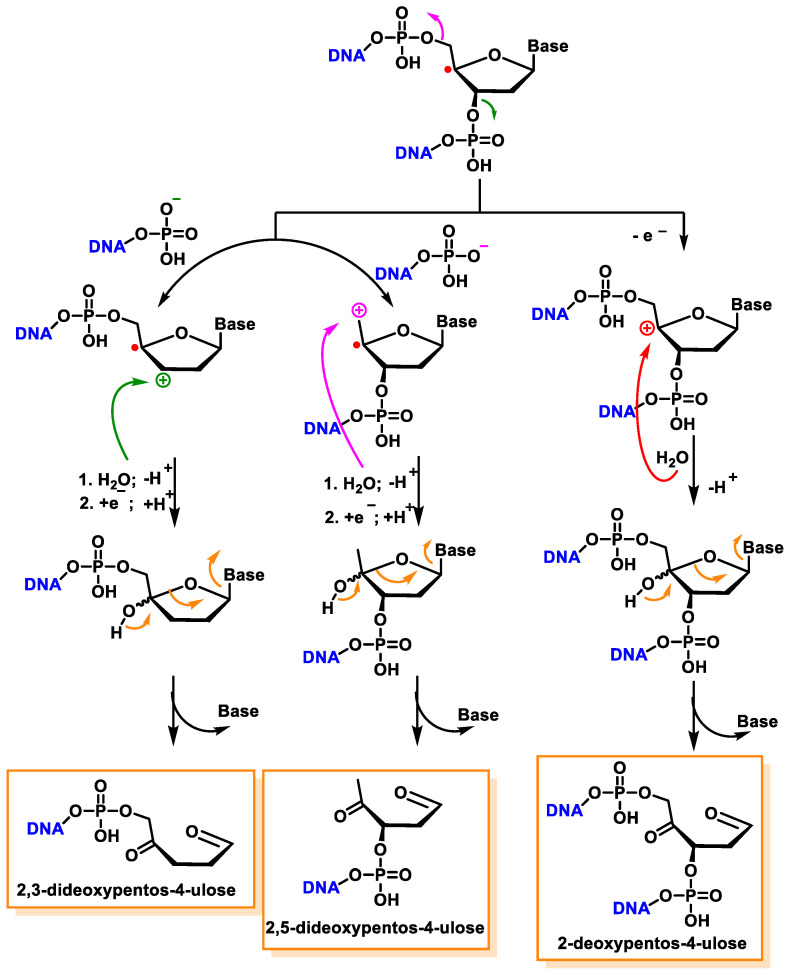
Mechanisms of product formation from reactions of the C4′ radical of 2′-deoxyribose.

Another pathway, independent of the presence of oxygen, exhibits that this radical readily loses the phosphate group by heterolytic cleavage of the phosphate at C3′ and C5′, the former being predominant over the latter, and this results in strand breakage and the formation of radical cations and the hydration of the radical cations, followed by the reduction and the release of the unaltered base, producing 2,3-didesoxypent-4-ulose and 2,5-didesoxypent-4-ulose as the final derivatives [147,148]. Oxidation of the C4′ radical without phosphate elimination forms a cation, leading to the formation of 2-deoxypentos-4-ulose upon the addition of HO, followed by the release of the unchanged base [149] (Figure 41).

In the presence of oxygen, the C4′radical produces a peroxyl radical, which is also the precursor of the 2-deoxypentos-4-ulose derivative, and neither 2,3-didesoxypentos 4-ulose nor 2,5-didesoxypentos-4-ulose is formed [44].

**Figure 42:**
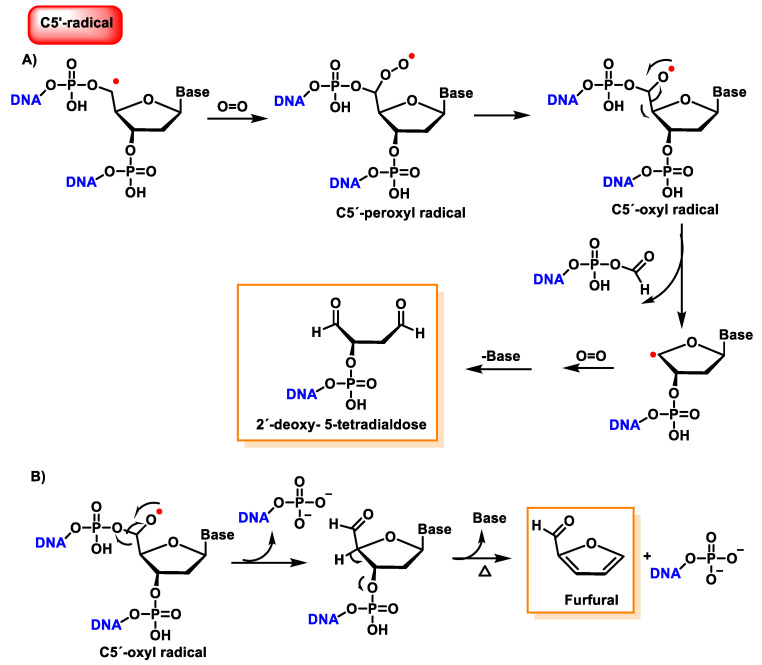
(**A**) Reaction mechanisms of the C5′radical of 2′-deoxyribose with O_2_, leading to 2′-deoxy- 5-tetradialdose formation. (**B**) Reaction mechanisms of the C5′radical of 2′-deoxyribose with O_2_, leading to furfural formation.

The C5′ radical, after oxygen treatment and removal of the base, produces 2-deoxytetradialdose as the final compound [147]. The hydroxylated C5′ position undergoes phosphate elimination to produce a 5′-aldehyde oligonucleotide, which subsequently by base and phosphate elimination generates furfural. This proposed reaction scheme is presented in part B of Figure 42.

**Figure 43:**
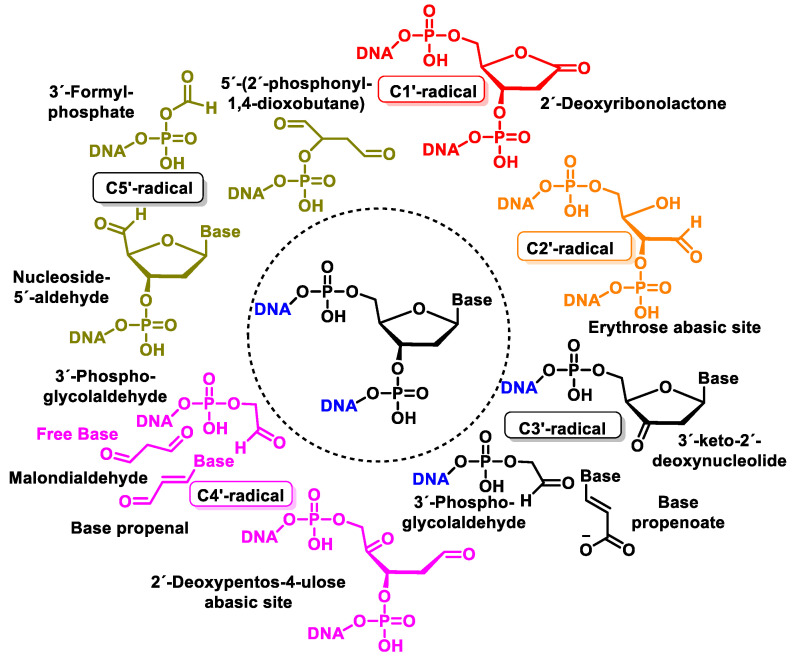
Structure of oxidation products of 2′-deoxyribose in DNA.

Figure 43 shows the products formed by the oxidation of 2′-deoxyribose in DNA [143]. Oxidation of the 2-deoxyribose moiety in DNA is also a determinant of the genetic toxicology of oxidative processes, which is involved not only in “strand breaks” but also in more complex DNA lesions, protein–DNA cross-links, and protein–DNA adducts.

## 11. Concomitant Damage to the Base and Sugar Moiety of the Same Nucleoside

### 11.1. 8,5′-Cyclopurine-2-deoxynucleosides

**Figure 44:**
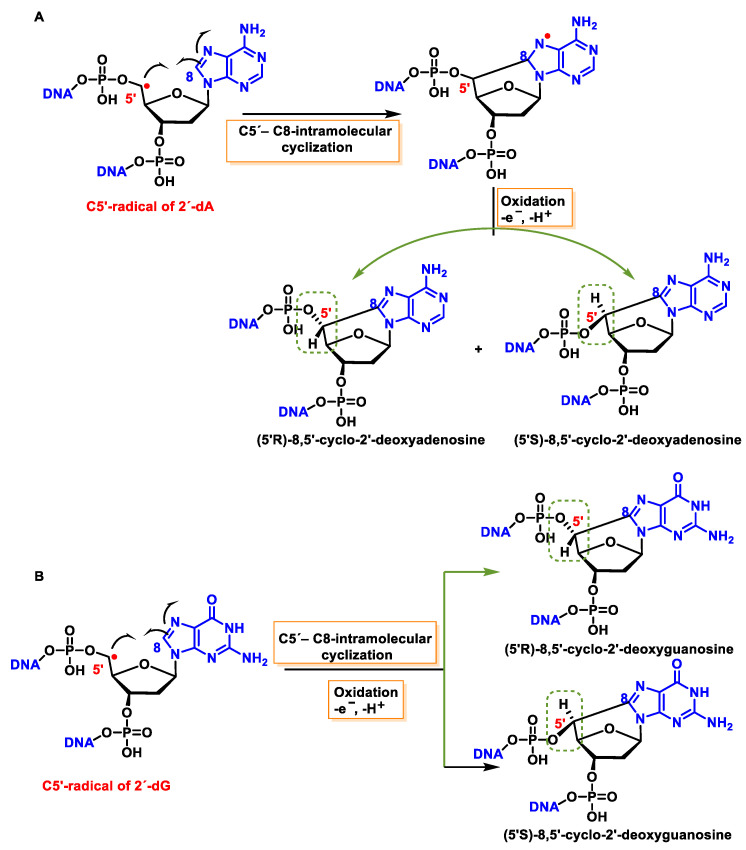
(**A**) Mechanisms of formation of (5′R)- and (5′S)-8,5′-cyclopurine-2′-deoxyadenosines and of (**B**) (5′R)- and (5′S)-8,5′-cyclopurine-2′-deoxyguanosines within DNA.

5′, 8-cyclo-2′-deoxynucleosides (cPu) of purine are tandem-type lesions observed among purine modifications of DNA and identified in mammalian cellular DNA in vivo. They are generated exclusively by ^•^OH attack on 2′-deoxyribose units, generating C5′ radicals, followed by cyclisation with the C8 position of the purine base. This is a highly stereo-specific attack on the C8 of the alanine and guanine ring within the purine nucleoside itself in the absence of O_2_, producing an intramolecular cyclisation between these two carbons and forming an N7-centred radical that is oxidized to produce 8,5′-cyclopurine-2′-deoxynucleosides, forming a new covalent bond between the C5′ and C8 positions. In this cyclisation, a new stereo-center is formed at C5′, and the reaction produces both 5′R and 5′S diastereomers of these compounds. The 5′, 8-cyclo-2′-deoxynucleosides (cPu) of purine are oxidative DNA lesions. This intramolecular cyclisation occurs because the C8 of the purines is particularly reactive towards radicals [134]. The mechanisms of formation of 8,5′-cyclopurine 2′-deoxynucleosides are shown in Figure 44.

These cyclized compounds represent the damage caused to both the base and the sugar moiety of the same nucleoside and are therefore considered tandem DNA lesions [150]. The rate constants for the intramolecular C5′-C8 cyclisation for the adenine derivative are 1.6 × 10^5^ s^−1^ and 1 × 10^6^ s^−1^ for the guanine derivative [151,152]. However, it has been observed that this reaction is inhibited when O_2_ is present at high concentrations, due to its ease of reaction with the C5′-centred radical [134,153].

### 11.2. Intrastrand Base–Base Tandem Lesions

In the absence of O_2_, an intracatenary cross-linking formation has been detected between the C8 of Gua and the CH_3_ group of Thy (Gu[8,5-Me]Thy).

In this lesion, a radical is formed on the methyl residue located on the CH_3_ of the thymine, and this allyl radical is the one that forms a covalent bond with the C8 carbon atom of the adjacent guanine base. The proposed mechanism for this cross-linking involves the addition of the allyl radical from Thy to the C8 of Gua, forming an N7 radical. These covalent cross-links, in addition to forming between the CH3 of Thy and the C8 of Gua, have also been observed between the C5 of Cyt and the C8 of Gua [154,155]. 

**Figure 45:**
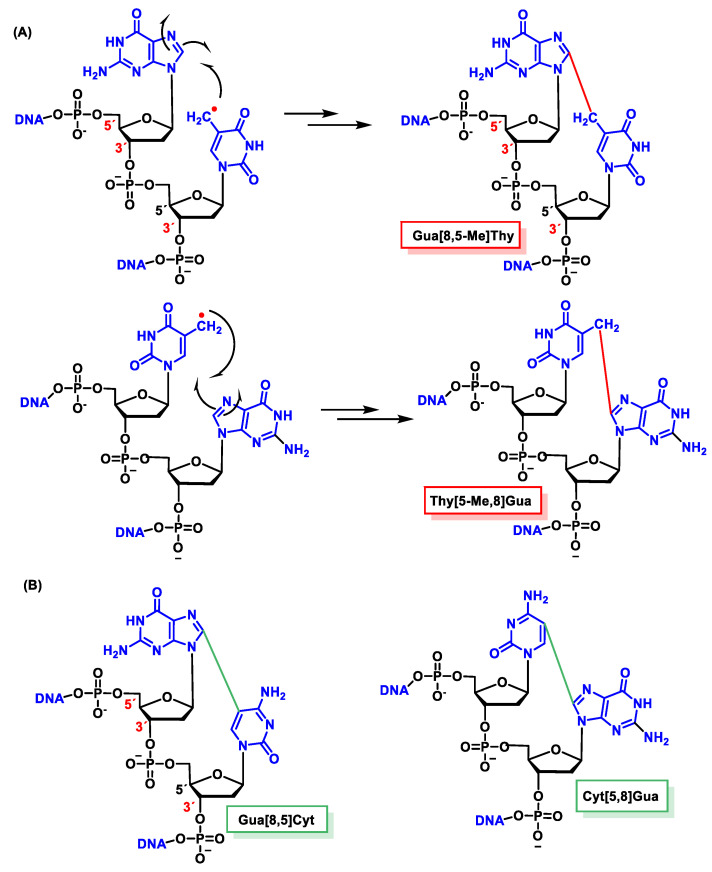
Structures of the intrastrand tandem lesions (**A**) Gua[8,5-Me]Thy and Thy[5-Me,8]Gua and (**B**) Gua[8,5]Cyt and Cyt[5,8]Gua.

The proposed formation mechanism of Gua[8,5]Cyt involves the addition of C6-OH-adducted Cyt radical to the adjacent C8 of Gua at the 5′ end forming a centered N7, followed by oxidation and dehydration. In subsequent studies, Gua[8,5-Me]Thy and an analogous Thy-Gua cross-link (Thy[5-Me,8]Gua) are formed in DNA, the former being generated in a higher yield than the latter, indicating that cross-linking is favored when the purine is at the 5′ end of the pyrimidine 2′ deoxynucleoside [156,157,158,159,160,161,162] (Figure 45).

Guanine–thymine tandem lesions were found to occur more efficiently than adenine-thymine cross-links. Ade-Thy (Ade[8,5-Me]Thy) and Thy-Ade (Thy[5-Me,8]Ade) cross-links have been identified in DNA irradiated with γ [163]. As in the case of Gua-Thy cross-links, Ade[8,5-Me]Thy was generated in a higher yield than Thy[5-Me,8]Ade. Gua[8,5-Me]Thy and cross-links between Gua and Cyt (Gua[8,5]Cyt) have been identified in gamma-irradiated live cells [164,165]. 

### 11.3. Interstrand Base–Base Tandem Lesions

**Figure 46:**
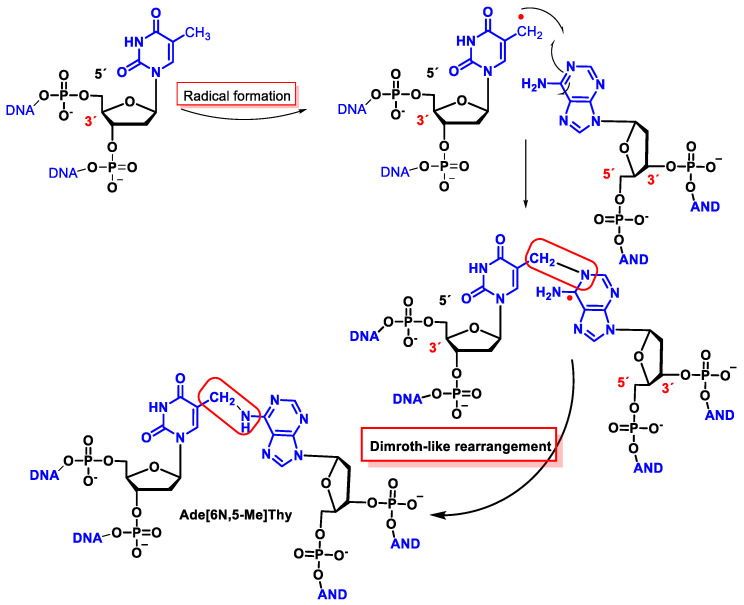
Structures of the interstrand tandem lesion Ade[6N,5-Me]Thy.

The ^•^OH forms an allyl radical from Thy by the loss of the hydrogen atom from the CH3 group of the thymine with the subsequent formation of a methylene radical on one strand and a cross-link with the amino group of Ade on the other exposed DNA strand [166,167,168,169]. The proposed mechanism has been performed using isotopic labelling and consists of the addition of the allyl radical to the cyclic Nen of Ade followed by a rearrangement, leading to a covalent bond between the CH_2_ of Thy and the 6-NH of Ade, following a Dimroth-like rearrangement [169,170] (Figure 46).

This interstrand cross-linking has been observed both in the presence and absence of O_2_, although the yield was lower in the latter case because O_2_ reacts with the allyl radical of Thy.

Lesion-like inter-intrastrand cross-linking or 5′,8-cyclo-2′-deoxypurines alter the internal or global helix geometry parameters, which are enforced by the presence of an additional covalent bond [171,172].

## 12. 8-Oxo-dG in DNA

**Figure 47:**
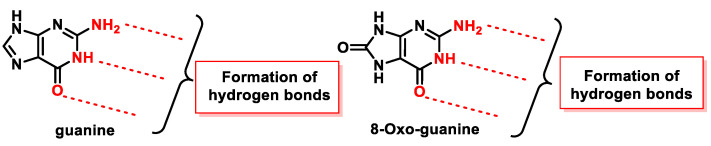
Groups that participate in the formation of hydrogen bonds in guanine and in 8-oxo-G.

8-oxodG is a lesion that guanine can undergo, characterized by the oxidation of the only carbon in the ring that has a hydrogen, carbon 8 of guanine, and the hydrogenation of the adjoining nitrogen to form a carbonyl with two adjacent amine groups. Figure 47 shows how the groups involved in the formation of hydrogen bonds in the Watson and Crick pairing are located in 8-oxo-G.

Alterations in the molecule, the presence of a carbonyl group, and the addition of one hydrogen to one of the nitrogens play a role in DNA replication. Prolonged presence of 8-oxoG in cells is lethal, as it is highly reactive and therefore easily degraded to several other stable products. In addition, elevated levels of 8-oxo-dG are frequently associated with carcinogenesis and other pathological states.

The amount of 8-oxo-dG produced is approximately 103/cell per day in normal cells, but this amount increases to 105/cell in cancer cells. The 8-oxopurines are more susceptible to oxidation than undamaged purines because the standard reduction potential of oxoG (0.74 V) and oxoA (0.92 V) are significantly lower than those of guanine (1.29 V) and adenine (1.42 V) [46].

8-oxo-G has a very low standard reduction potential (E° = 0.74 V compared to 1.29 V for guanine) which makes it susceptible to further oxidation and thus other alterations of the nitrogenous base [117]. 8-oxo-dG is highly mutagenic due to its propensity to pair with adenine in a syn conformation, resulting in a guanine to thymine mutation [173].

DNA polymerase β (pol β) accommodates the 8-oxo-dG template in the syn conformation, thereby incorporating adenine into the replication chain. 8-oxo-dG can be formed not only in DNA molecules but also in free nucleotide, whose pools are particularly vulnerable to oxidative damage (8-oxo-dGTP) [174]. As 8-oxo-dGTP causes changes in the active site of pol β, its synla conformation can be inserted into the opposite adenine, avoiding recognizing it as damaged, thus resulting in an A:C (same as T:G) mutation called 8-oxoguanine [175]. 8-oxoguanine was first discovered in DNA during the characterization of cancer-causing molecules related to oxidative stress; therefore, it has been widely used as a biomarker of ROS, but also of oxidative stress, mutagenesis, and carcinogenesis levels [176,177].

The critical feature of 8-oxoguanine is that its syn conformation uses a Hoogsteen edge to pair bases with adenine, while its anti-conformation still pairs with cytosine as an unoxidized guanine. Thus, 8-oxo-dG causes guanine to thymine transversion, leading to mutations [178], especially in cancer [179]. Structural analysis has revealed that 8-oxoG can adopt either an anti or syn conformation.

**Figure 48:**
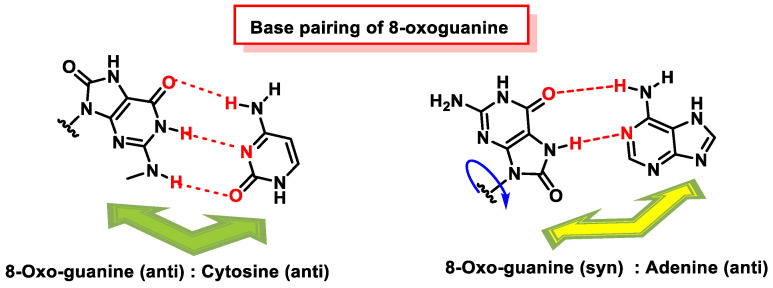
Base pairing of 8-oxoguanine. 8-oxoguanine pairs with cytosine (C) through its anti-conformation. The 8-oxoguanine in syn conformation uses a Hoogsteen edge to pair with adenine.

The reason why 8-oxoguanine causes cytosine–adenine substitutions, when the Watson and Crick bonding atoms have not been altered, is because this substitution occurs on the basis of Hoogsteen pairing (Figure 48). In the Watson and Crick pairing, both nitrogenous bases are bonded to the so-called anti face, but by rotation of the N-glycosidic bond, a nitrogenous base can be bonded on another face, the syn face. A Hoogsteen pairing occurs between a nitrogenous base in the anti position and one in the syn position. In the case of the Hoogsten pairing, the 8-oxoG is different from the guanine and can bind to the adenine. Protein polymerase II can mistakenly introduce an adenine due to this type of pairing, and this replication error commonly evades the usual DNA error correction mechanisms [180,181].

8-oxoG in the anti-conformation forms Watson–Crick base pairs with cytosine, whereas the lesion in the syn conformation uses the Hoogsteen edge of the lesion to form a base pair with adenine. DNA polymerases incorporate adenine in addition to cytosine against 8-oxoG [173,182,183], and this induces a G:C-T:A transversion in *Escherichia coli* [184] and mammalian cells [185].

8-oxoguanine is a lesion responsible for cytosine to adenine substitutions, and on further replication, the guanine is substituted by a thymine, first due to Hoogsten pairing and later due to normal replication of the complementary strand formed. Such substitution is relatively frequent, with 10–75% of 8-oxo-G substitutions, resulting in the cytosine-to-guanine substitution becoming a source of mutations for the cell [54]. In *E. coli* bacteria, 8-oxo-G also appears to cause adenine-to-cytosine substitution, but such a substitution does not occur in mammals.

## 13. Major DNA Repair Pathways

**Figure 49:**
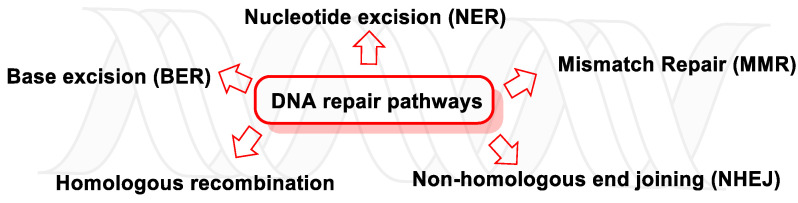
DNA repair pathways.

With regard to the hydrolytic stability of the DNA bonds, the most labile under physiological conditions is the N-glycosidic bond. Any modification of the nitrogenous bases of DNA, such as oxidation by ROS/RNS, can hydrolyze the N-glycosidic bond, thus cleaving the nitrogenous base from the deoxyribose and leaving an apurinic/apyrimidinic (AP) site [186]. There are five main DNA repair pathways, but if damaged DNA persists, apoptosis is triggered so that tissues can get rid of cells with an unstable genome (Figure 49).

Single-strand breaks (SSBs) are repaired by nucleotide excision, base excision, and mismatch repair (NER, BER, and MMR, respectively) and are usually generated by ROS damage, abasic sites, or erroneous DNA topoisomerase 1 (TOP1) enzyme activity [187]. Unresolved SSBs can collapse DNA replication, paralyze ongoing transcription, and trigger the polyADP-ribose polymerase 1 (PARP1, mostly present in cell nucleus and involved in normal or abnormal recovery from DNA damage), which releases cellular NAD+, ATP, and apoptosis-inducing factor (AIF) [188].

In many cancers, the DNA damage response (DDR) and DNA damage tolerance pathways are deregulated, leading to increased genomic instability, mutagenesis, and neoplastic progression [189].

### 13.1. DNA Damage Response (DDR)

When DNA is altered, a series of sensor proteins initiate a repair response, i.e., they detect damage, signal its presence, and promote repair, attempting to eliminate the damage in a substrate-dependent manner [190], through five major repair pathways (which are active throughout different stages of the cell cycle): BER, NER, MMR, homologous recombination (HR), and non-homologous end joining (NHEJ). These repair processes are key to maintaining the genetic stability of cells [6].

#### 13.1.1. Base Excision Repair (BER)

BER maintains the integrity of the genome and prevents premature ageing, cancer, and other human diseases by removing small, non-helix-distorting base or bulky helix-distorting lesions [191]. BER repairs thousands of DNA lesions and strand breaks caused continuously by endogenous and exogenous mutagens [192]. Its function requires strict and continuous control of the availability of the building blocks necessary for rapid and accurate repair and spatial and temporal coordination with cell cycle progression to prevent replication of damaged DNA [193]. Examples of BER-repaired base lesions include adenine incorporation via 8-oxoguanine during DNA replication; the oxidized bases are 8-oxoguanine and 2,6-diamino-4-hydroxy-5-formamidopyrimidine (FapyG, FapyA); the alkylated bases are 3-methyladenine and 7-methylguanosine; the deaminated bases are hypoxanthine formed from adenine deamination (xanthine formed from guanine deamination) and uracil inappropriately incorporated into DNA or formed by cytosine deamination [194]. The defect in the BER pathway leads to predisposition to cancer by producing an increased mutation rate, and somatic mutations in Pol β have been found in 30% of human cancers, and some of these mutations lead to cancer transformation when expressed in mouse cells [195]. In short, BER removes bases damaged by oxidative or alkylating patterns or inappropriate bases generated endogenously or induced by genotoxicants [187].

#### 13.1.2. Nucleotide Excision Repair (NER)

The NER, in mammals, is the main pathway for the elimination of bulky lesions, such as those induced by ultraviolet light, environmental mutagens, and some cancer chemotherapeutics, which produce voluminous DNA adducts. The discovery of NER, its mode of action, and relationship to other cellular pathways have been extensively reviewed in several articles on DNA repair and mutagenesis [6]. Recognition of the formed adducts (mainly thymine dimers and 6,4-photoproducts) leads to the elimination of the short segment of single-stranded DNA containing the lesion (the undamaged one remains and DNA polymerase uses it as a template to produce a short complementary sequence). The final ligation is performed by DNA ligase, which completes the NER and forms a double-stranded DNA [196].

NER dysfunction results in DNA polymorphism. Studies have shown that polymorphisms in exon 10 (G>A) (Asp312Asn) and exon 23 (A>T) (Lys751Gln) are associated with genetic predisposition to various types of cancer [197,198]. There are several genes encoding proteins of the NER pathway, such as XPC and XPD. In a study on relapse rates of high-risk stage II and III colorectal cancers, the 2251A>C polymorphism of XPD (ERCC2) was significantly correlated with early relapse after chemotherapeutic treatment [199]. According to Gorbunova et al., NER presence in different cells and tissues have been correlated to a decrease in NER capacity with age, which may be due to reduced constitutive levels of proteins used in the NER pathway [200].

#### 13.1.3. Mismatch Repair (MMR)

MMR is a strand-specific, post-replicative repair pathway used for the recognition and the restoration of insertion, deletion, and base misincorporation errors that occur during DNA replication and recombination [201]. The synthesis of a DNA strand often contains errors and, in order to initiate repair, the procedure must recognize the mismatches originating from the original template. It is suspected that, in eukaryotes, newly synthesized DNA transiently contains notches (before being sealed by DNA ligase) and provides a signal that directs mismatch correction systems to the appropriate strand, so these notches must be present on the leading strand [202]. Mismatches are usually due to the tautomerization of bases during DNA replication, and examples of mismatched bases include a G/T or A/C pairing [203]. The damage is repaired by recognizing the deformity caused by the mismatch, comparing the template and synthesized strands and removing the mis-incorporated base and replacing it with the correct nucleotide. From a few to thousands of base pairs can be removed from the newly synthesized DNA strand [204]. To date, several human MMR genes have been identified (MLH1, MLH3, MSH2, MSH3, MSH6, PMS1, and PMS2, which coordinate sequentially to initiate DNA repair). Their mutations generate an acute defect in repair and lead to the progressive accumulation of alterations throughout the genome and may result in a hereditary predisposition to non-polyposis colorectal cancer, ovarian cancer, and other types of cancer [205].

#### 13.1.4. Homologous Recombination (HR)

The homologous recombination (HR) mechanism comprises a series of interrelated pathways that repair DNA double-strand breaks (DSBs) and interstrand cross-links (ICLs).

In ICL, two complementary chain bases are covalently linked due to the action of cross-linking agents, such as cisplatin compounds, nitrogen mustards (cytotoxic organic compounds with a chloroethylamine functional group), MMC (Mitomycin C, a quinone-containing antitumor drug), psoralens (widely used in the treatment of psoriasis, eczema, vitiligo, and cutaneous T-cell lymphoma), and alkylating agents [206], but also by environmental exposures and cancer chemotherapeutic agents with two reactive groups [207]. ICLs prevent the separation of two DNA strands and therefore end up interfering with DNA replication and transcription, which are essential cellular processes. In ICL repair, both strands of DNA must be cut to completely remove the lesion, so it is a sequential process that avoids double-strand breaks [208]. Inadequate ICL repair is particularly detrimental to rapidly dividing cells, which explains the bone marrow failure characteristic of Fanconi anemia and why cross-linking agents are effective in cancer therapy [209]. ICL genes have been found to have a strong association with cancer, and several of which are involved in hereditary breast cancer and ovarian cancer syndrome (HBOC) [6].

Eukaryotic cells have evolved several repair pathways to repair DSBs by homologous recombination (HR) and non-homologous end-joining (NHEJ), which can induce small-scale mutations and chromosomal aberrations [210]. Double-strand breaks (DSBs), which are one of the most harmful and mutagenic forms of DNA damage, are induced by various chemical and physical agents (such as ionizing radiation, but also occur spontaneously during cellular processes and even at a high frequency) and are considered critical lesions in the formation of chromosomal aberrations [211]. DSBs defects in the two pathways involved (HE or NHEJ) cause genome instability and promote tumorigenesis, inducing cell death or a wide variety of genetic alterations.

#### 13.1.5. Non-Homologous end Joining (NHEJ)

NHEJ is a pathway that repairs DSBs in DNA. NHEJ is termed “non-homologous” because the break ends are joined without the need for a homologous template. The term “non-homologous end joining” was coined in 1996 by Moore and Haber [212]. NHEJ is often guided by microhomologies (composed of short homologous DNA sequences), which are usually present in single-stranded overhangs at the ends of double-strand breaks [213]. When the overhangs are compatible, NHEJ repairs the break accurately. If the repair is inaccurate, nucleotide loss results, a common loss that is observed when the overhangs are not compatible. Inaccurate NHEJ can lead to translocations and telomere fusion, a features of tumor cells [214]. When the NHEJ pathway is inactivated, double-strand breaks can be repaired by a more error-prone pathway called microhomology-mediated end joining (MMEJ), in which end resection reveals short microhomologies on either side of the break, which guide the repair [215]. This is in contrasts with the classical NHEJ, as MMEJ repair results in the elimination of the DNA sequence between the microhomologies.

In the NHEJ pathway, the p53-binding protein 1 (53BP1) plays an important role in recruiting the components (the order of recruitment depends on the complexity of the DNA damage [216]) to the site of cleavage, and activates signaling and facilitates the synapse of the two ends [217].

### 13.2. DNA Damage Tolerance Pathways (DDT)

Mutations are not necessarily harmful and can be beneficial under certain conditions, for example, when genetic diversity is advantageous, such as somatic hypermutation and antibody generation [218]. Mutations in DNA are caused by deficient repair of lesions generated by harmful agents, since the genome is particularly vulnerable to this damage [219]. Lesion circumvention is accomplished by a set of error-prone and error-free processes collectively referred to as DNA damage tolerance mechanisms [220]. Although the above mechanisms can repair different types of DNA lesions, it is likely that the replication machinery will still encounter poorly repaired or even unrepaired lesions, and the replication of a damaged genome results in a high frequency of collapse and genome instability [221]. In these circumstances, cells employ an alternative pathway called the DNA damage tolerance (DDT). DDT is not a repair pathway per se, but provides a mechanism to tolerate DNA lesions, thereby increasing survival and preventing genome instability. Paradoxically, DDT is also associated with increased mutagenesis, which can lead to cancer development, making DDT a double-edged sword in genome protection [220].

DDT favors the circumvention of single-stranded DNA lesions encountered by DNA polymerases during DNA replication. Two mechanistically different branches of DDT have been characterized are as follows: translesion synthesis (TLS) and template switching (TS) [222].

TLS, a conserved mechanism from bacteria to mammals, is mechanistically simple and straightforward, but error-prone, as it is carried out by specialized TLS polymerases, which can replicate DNA lesions with less fidelity than replicative DNA polymerases [222]. Eleven TLS polymerases are known (REV1, POL η, POL ι, POL κ, POL ζ, POL μ, POL λ, POL β, POL ν, and POL θ) that are distributed in four families (Y, B, X, and A) and PrimPol [223]. If TLS polymerases were to incorporate incorrect nucleotides, in the next round of replication, they would result in mutations, leading to tumorigenesis and disease, but at the same time, they can contribute to the evolution of organisms [224]. The frequency of error occurrence during TLS depends on several factors, such as the biochemical characteristics of the TLS polymerase and the context of the DNA sequence [225]. TLS polymerases, which are required for ICL repair, may also play a role in the BER and NER pathways to obtain new DNA [226], and REV1 and REV3 polymerases have been found to be involved in the development of chemoresistance in human cells, which may pave the path of a new class of chemotherapeutic drugs [227].

In the template switching mechanism (TS, a complex but preferable process to avoid DNA lesions), the stalled nascent strand changes from the damaged template to the newly synthesized and undamaged sister strand [220]. TS is a form of error-free DNA damage tolerance that creates complementary reverse copies of sequential regions by replicating along the complementary or nascent DNA strand, producing a reverse repeat capable of building the stem of a perfect DNA hairpin or fixing the base pairing of an existing stem [228]. TS is normally thought to trigger large structural changes, and this mechanism can explain complex sequence changes, although structure-breaking mutations are rarely fixed in evolution [229].

## 14. DNA Oxidation as a Strategic Antimicrobial Mechanism

In response to the challenge of antibiotic resistance, researchers have focused their efforts on developing viable antibacterial methods [230]. The use of ROS has been proposed as a promising antibacterial approach to eradicate pathogenic microorganisms [231]. Evidence of ROS-mediated antimicrobial lethality was first provided by Kohanski et al. in 2007 [232]. The occurrence of highly deleterious oxidative radical species, particularly ^•^OH, may play a role in cell death induced by norfloxacin, ampicillin, and kanamycin in *E. coli* [233]. The formation of ^•^OH is influenced by the depletion of NADH, which is associated with the cellular metabolism as well as the tricarboxylic acid (TCA) cycle, the electron transport chain, the damage of iron–sulfur clusters in proteins, and the activation of the Fenton reaction [234]. Another hypothesis proposed by Hong et al. states that ROS-mediated damage, secondary to the main lesion and caused by stress, can trigger a self-reinforcing ROS accumulation and subsequently lead to a self-directed death process in bacteria [235].

Antimicrobial peptides (AMPs) typically work by targeting the membranes of microbial cells, disrupting their integrity and leading to cell death [236,237]. However, some AMPs may interact with DNA, which could potentially result in DNA damage. These interactions can lead to DNA strand breaks, cross-linking, and other modifications that can compromise the integrity and functionality of the DNA molecule. The accumulation of DNA damage and the disruption of cellular processes can ultimately lead to cell death [238]. If the oxidative damage to DNA is severe and the cell cannot repair it properly, it can result in genetic mutations, loss of cell viability, and reduced ability for the microbe to replicate and cause infection [239,240]. AMP-17, an antimicrobial peptide from the housefly (*Musca domestica*), is widely known for its potent inhibitory properties against many fungal infections, particularly *Candida albicans* [241]. Administration of AMP-17 led to an increase in the concentration of reactive oxygen species (ROS), depolarization of mitochondrial membrane potential (MMP), and changes in the cell cycle culminating in apoptosis and necrosis, thus promoting the death of *C. albicans* cells [241]. Similarly, other AMPs, such as LL-37, PMAP-23, cecropin, and pleurocidin, were found to induce apoptosis in *C. albicans* by oxidative stress [242,243,244,245]. The overproduction of mitochondrial ROS disrupted the intracellular redox homeostasis in *C. albicans* and reduced the glutathione level in fungal cells, resulting in oxidative stress. The introduction of small compounds that can regulate antioxidant levels and/or intracellular reactive oxygen species (ROS) has the potential to disrupt the cellular oxidative milieu and trigger cellular apoptosis. Vitamin C and GSH-glutathione, two antioxidants, were able to decrease the amount of active oxygen species induced by cecropin [244].

## 15. Methods for DNA Damage Assessment

**Figure 50:**
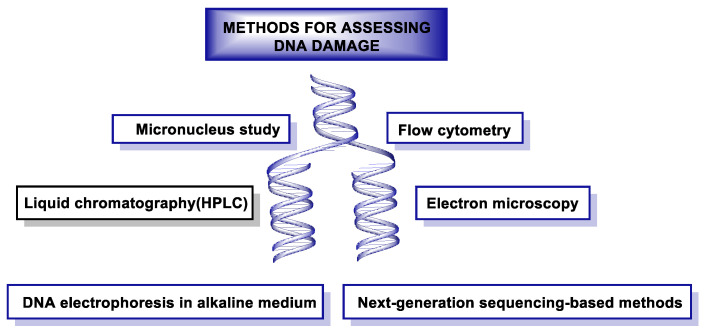
Methods used to assess DNA damage.

DNA damage can be measured using different methods shown in Figure 50.

In recent decades, a number of methods have been developed to detect damage in DNA. One of the techniques used for this monitoring is the single-cell gel electrophoresis assay or “Comet assay”, which makes it possible to assess the damage caused to genetic material by different chemical and physical agents. This technique was developed in the mid-1980s [246,247]. Immunoassay-based methods were also released in the same decade, such as radioimmunoassay (RIA) [248], enzyme-linked immunosorbent assay (ELISA) [249], and immunoblotting [249], which are used to quantify DNA damage induced by UV radiation. In the same decade, but especially in the early 1990s, polymerase chain reaction (PCR) methods [250], such as quantitative PCR (qPCR) [251]and ligation-mediated PCR (LMPCR) [252], were used to map DNA damage at nucleotide resolution. In 1992, the TUNEL (TdT-mediated dUTP-biotin Nick End Labeling) assay was used to label DNA breaks in situ to study apoptosis [253]. In 2011, a method combining immune precipitation and microarray was developed to identify DNA damage at the genomic scale in yeast [254], and in 2014, at the chromosomal scale in humans [255]. In the last five years, new methods based on next-generation sequencing (NGS) have emerged to detect DNA damage and repairs with nucleotide resolution [256].

These NGS-based methods have provided clinicians and researchers with the ability to accurately locate genome-wide damage. Thirdgeneration (so-called “long-read”) sequencing technologies are capable of detecting ribonucleotide incorporation and UV-induced DNA damage [257].

### 15.1. Gel Electrophoresis-Based Methods

Gel electrophoresis is a simple and efficient way to separate DNA fragments, ranging from 50 bp to 500,000 bp, and DNA strand breaks and other types of DNA damage can be analyzed under denaturing conditions [258]. The comet assay also relies on gel electrophoresis to detect chain breaks on a microscope slide [247]. This method gained popularity after its development because of its simplicity and sensitivity for detecting global DNA damage and repair, but it does not provide information on individual genes. The combination of the comet method with fluorescence in situ hybridization (FISH) (Comet-FISH) enables the observation of specific genes [259].

Another problem is the great variability between samples and their laboriousness for genotoxicity screening. A newer technology, called CometChip, overcomes these limitations by using vertical micromoulded casting cassettes in a 96-well agarose gel. Each well has about 500 microwells, and each well can accommodate a single cell [260,261]. This is useful for high-throughput genotoxicity testing and measurement of DNA damage and repair at the general level of strand breaks, but it does not provide information on the location of damage and repair [262].

### 15.2. Radioactive-Based Methods

In recent years, the radioactive method has been widely applied due to its high sensitivity, combined with other techniques, such as immunoassay and PCR. However, these methods are very laborious and have to be conducted under strict radioactive safety conditions [263]. PCR amplification and radioactive end-labelling are commonly used to isolate and recognize specific genes. The Southern blot assay measures the frequency of DNA lesions with radioactive probe hybridization after alkaline gel electrophoresis [264], but it cannot achieve nucleotide resolution. High-resolution, single-nucleotide techniques are called LMPCR (ligation-mediated PCR), single-strand ligation PCR (sslig-PCR), and primer extension, which aim to amplify the signal [265,266].

### 15.3. Fluorescence-Based Methods

Fluorescence-based methods, combined with fluorescence microscopy and flow cytometry, are widely used because of their high sensitivity, specificity, and ease of use. However, they also have some limitations, i.e., they do not provide genomic sequence information and the photochemical destruction of fluorophores is a drawback during quantitative analysis [267,268].

### 15.4. Next-Generation Sequencing-Based Methods

Determining the distribution of DNA damage at nucleotide resolution is critical for characterizing genotoxicity and analyzing exposure to specific environmental carcinogens [269]. Although several radioactive methods with single-nucleotide resolution have been developed, the problem associated with them is that they are limited to small regions of the genome. Mass spectrometry-based methods are very useful for the identification and quantification of DNA adducts, but they cannot provide genome sequence information [270,271].

Next-generation sequencing (NGS) technology provides low-cost, high-throughput DNA sequencing data with high accuracy and can quantify genome-wide damage [272]. This technology involves three main strategies: (i) immunoprecipitation or capture with biotin–streptavidin to enrich fragments carrying DNA damage, (ii) the second approach, enzymatically or chemically, creates a nick at the damage site and ligates the sequencing adapter for NGS library preparation, and (iii) the third strategy is based on immunoprecipitation and high-fidelity DNA polymerase arrest prior to the lesion, to magnify the DNA damage and localize its position [273].

In addition to those mentioned above, there is another strategy that uses in situ end labeling to detect DSBs [274], and the following techniques are based on this strategy: BLESS (Breaks Labeling, Enrichment on Streptavidin and next-generation Sequencing), END-seq, BLISS (Breaks Labeling In Situ and Sequencing), and i-BLESS (immobilized-BLESS) [275,276,277,278].

For SSB detection, two new methods are also available: SSB-seq and SSiNGLe (single-strand break mapping at nucleotide genome level) [279,280].

## 16. Conclusions

Living organisms are continuously exposed to a wide variety of damaging agents, such as free radicals, which can modify DNA and even induce disease. DNA damage refers to any modification in the DNA structure that alters its coding properties and/or interferes with cellular processes. Reactive stress (which encompasses damage caused by ROS and RNS) causes DNA damage in a variety of ways, including base modifications, abasic sites, and strand breaks. Oxidative DNA damage, such as 8-oxo-dG, may contribute to carcinogenesis through two mechanisms: modulation of gene expression or through induction of mutations. The endogenous species of ROS are O_2_^•−^, ^•^OH, HO_2_^•^, H_2_O_2_, RO_2_^•^, and HOCl, and those of RNS are NO^•^, NO_2_^•^,ONOO^−^, HNO_2_, RONOO, and N_2_O_3_. This review elaborates in some detail the chemistry involved in the modifications caused by superoxide anion, singlet oxygen, hydroxyl radical, and peroxynitrite.

To counteract the effects caused by ROS and RNS, living organisms have robust DNA repair mechanisms, eliminating or tolerating damage to ensure overall survival. Deviations in this adjustment destabilize cellular metabolic homeostasis, which may induce cancer by causing genomic instability. This review has outlined the mechanisms of DNA damage and the repair/tolerance pathways that counteract them, thus providing a comprehensive chemical overview of this issue.
